# S‐Nitrosothiols as Thiol‐Protecting Groups for Controlled Thiol‐Maleimide Crosslinking of Homogeneous Soft Hydrogels

**DOI:** 10.1002/adma.74627

**Published:** 2026-08-17

**Authors:** Julian A. Serna, Michelle J. Iwohn, Maximilian Seifermann, Stefan Heißler, Maike Schliephake, Franziska Dopp, Claudia Muhle‐Goll, Pavel A. Levkin

**Affiliations:** ^1^ Institute of Biological and Chemical Systems‐Functional Molecular Systems (IBCS‐FMS) Karlsruhe Institute of Technology (KIT) Kaiserstrasse 12 Karlsruhe Germany; ^2^ Institute of Functional Interfaces (IFG) Karlsruhe Institute of Technology (KIT) Hermann‐von‐Helmholtz‐Platz 1 Eggenstein‐Leopoldshafen Germany; ^3^ Institute for Biological Interfaces 4 (IBG‐4) Karlsruhe Institute of Technology (KIT) Kaiserstrasse 12 Karlsruhe DE Germany; ^4^ Institute of Organic Chemistry (IOC) Karlsruhe Institute of Technology (KIT) Kaiserstrasse 12 Karlsruhe DE Germany

**Keywords:** 3d cell culture, hydrogel, Michael´s addition, maleimide, polyethylene glycol, polymer chemistry, polymer, s‐nitrosothiols, thiol

## Abstract

The thiol‐maleimide Michael‐type addition is used in bioconjugation and hydrogel crosslinking for its chemoselectivity and rapid kinetics under physiological conditions. However, this same reactivity limits its use for soft hydrogels, as gelation often proceeds faster than precursor mixing, leading to spatially heterogeneous networks. Here, S‐nitrosothiols (RSNOs) are introduced as thiol‐protecting groups that suppress premature thiol‐maleimide coupling and enable homogeneous mixing of polymer precursors prior to on‐demand crosslinking. Despite common assumptions about RSNO instability, RSNO‐modified 4‐arm polyethylene glycol (PEGSNO) is stable in aqueous solution for months at 4°C protected from light. The nucleophiles sodium ascorbate or sodium thiosulfate (STS) trigger thiol regeneration and controlled crosslinking with maleimide‐modified PEG. The resulting hydrogels are homogeneous with Young's moduli tunable across a physiologically relevant range by independently varying nucleophile identity, concentration, or polymer content. In a cell‐adhesive system based on maleimide‐modified cold water fish skin gelatin, STS‐triggered gelation supports 3D cell encapsulation, with cytocompatibility depending on formulation and cell type, while matrix stiffness is tuned through polymer content and STS concentration. This work establishes RSNO chemistry as a previously unexplored strategy for controlled thiol‐maleimide crosslinking, enabling reproducible, homogeneous network formation, with potential for dual‐function materials coupling network formation with local reactive nitrogen species delivery.

## Graphical abstract

Rapid thiol‐maleimide addition frequently outpaces mixing, resulting in heterogeneous hydrogels. S‐nitrosothiols act as thiol‐protecting groups, allowing uniform mixing with maleimide‐functionalized polymers before gelation. Sodium thiosulfate or sodium ascorbate then regenerates thiols on demand, triggering controlled thiol‐maleimide crosslinking. This strategy yields homogeneous hydrogels with tunable mechanics that support cell encapsulation, while releasing reactive nitrogen species as they form.

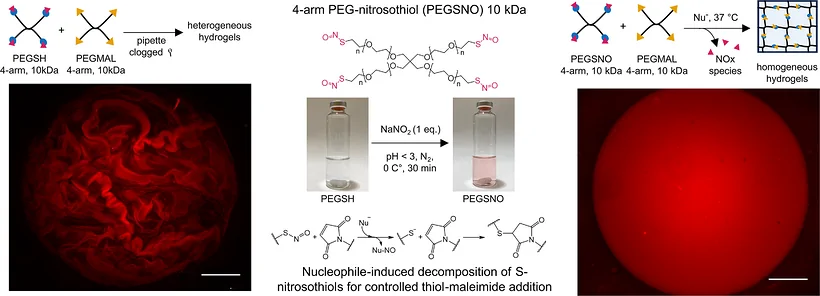

## 1 Introduction

Embedding cells in soft, three‐dimensional scaffolds made from synthetic or natural polymers has enabled scientists to recapitulate key biochemical and mechanical features of the native extracellular microenvironment in vitro. Hydrogel‐based scaffolds are now central tools in fields such as mechanobiology, tissue engineering, disease modeling, organoid research, and biofabrication, where independently tunable matrix properties can be used to influence or direct cell fate and function.

Central to the design of cell‐instructive hydrogels is the chemistry used to crosslink the polymer network, as it governs not only the conditions under which gelation occurs (and therefore the cytocompatible gelation window), but also the mechanical and physical properties of the resulting network. Among the many hydrogel crosslinking strategies developed to date, which include photo‐induced free‐radical polymerization, dynamic covalent chemistries, and supramolecular assembly, step‐growth reactions based on thiol chemistry, have emerged as particularly attractive for cell encapsulation applications [1]. This is due to their ability to react selectively with diverse functional groups under mild, aqueous and catalyst‐free conditions. The thiol‐maleimide Michael addition is particularly valued in bioconjugation for its high chemoselectivity and rapid kinetics at neutral pH [2], and has been used in biomaterials research to generate injectable matrices [3], mechanically tunable scaffolds [4] and well‐defined 3D environments for studying single cell‐matrix interactions. Despite forming stable covalent bonds, recent works have shown the susceptibility of thiosuccinimide crosslinks to photodegradation [5] and the possibility to perform post‐crosslinking modifications on networks with free pendant maleimides [6]. Moreover, maleimides can participate in diverse network‐forming chemistries, like photoinduced [2+2] homocycloaddition, thermally‐allowed [4+2] cycloaddition with dienes (Diels‐Alder reaction), and radical polymerization, among others [7]. This highlights the considerable yet largely unexplored potential of maleimides in soft polymer chemistry and their promise for the design of multi‐network materials with advanced properties [6, 8].

The rapid kinetics that makes the thiol‐maleimide chemistry so efficient and useful in bioconjugation is, paradoxically, its main limitation when using it for cell‐embedded soft hydrogels. Under physiological temperature (37°C) and pH (∼7.4), the reaction often proceeds faster than the timescale of manual or mechanical mixing of the precursors [9], thus inevitably leading to spatially heterogeneous networks on both the micro‐ and macro‐scale. These heterogeneities produce gradients in network mechanics and small‐molecule diffusion, which compromise reproducibility and confound the biological interpretation of cell culture experiments. To overcome the heterogeneity problem, several strategies, such as (i) increasing the pKa of the thiol to slow down the reaction by carefully selecting the amino acids before the cysteine in peptide linkers [10, 11], (ii) performing the reaction at acidic pH for slower gelation kinetics [9, 12], or (iii) using coumarin‐based photocages to deprotect the thiols on demand [13] have been developed. While effective in specific settings, each approach carries inherent limitations: pK_a_ engineering is not readily applicable beyond peptide‐based linkers, acidic pH is harmful to cells, and photo cages are difficult to synthesize and introduce bulky moieties that can sterically interfere with network architecture. A more recent strategy used host–guest chemistry between β‐cyclodextrin (β‐CD) and maleimide groups to slow down the thiol‐maleimide crosslinking kinetics and improve network homogeneity in poly(ethylene glycol) (PEG)‐based hydrogels [14]. However, this approach lacks an on‐demand trigger, operates within a narrow β‐CD concentration window, outside of which network defects increase, and requires a post‐gelation dialysis step (to remove the cytotoxic β‐CD), that is incompatible with cell encapsulation. Broadly applicable and biocompatible strategies to control thiol‐maleimide reaction kinetics in cell‐embedded hydrogels without sacrificing ease of use remain elusive.

Here, we address this challenge by introducing S‐nitrosothiols (RSNOs) as a new class of thiol‐protecting groups for controlled polymer network formation in soft hydrogels. RSNOs are well established in biomedical research as nitric oxide (NO) carriers and signaling intermediates [15, 16], and polymer‐ or nanoparticle‐bound RSNOs [17, 18, 19, 20] have been explored as NO‐releasing platforms for therapeutic applications in tissue regeneration. However, to the best of our knowledge, RSNOs have not been exploited as functional protecting groups in polymer network formation despite their rich chemistry. In this work, we demonstrate that conversion of thiols into RSNOs effectively suppresses their nucleophilicity, thereby preventing premature thiol‐maleimide coupling and enabling homogeneous mixing of polymer network precursors prior to crosslinking. We synthesize and characterize RSNO‐modified PEG (PEGSNO) and show that, contrary to common assumptions about RSNO instability, these materials exhibit remarkable long‐term stability in solution under appropriate conditions. We evaluate sodium ascorbate (SA) and sodium thiosulfate (STS) as two mild, cytocompatible nucleophiles in their ability to trigger on‐demand regeneration of free thiols from PEGSNO, thus initiating controlled thiol‐maleimide crosslinking and yielding homogeneous hydrogel networks. Through mechanistic studies, rheological characterization and spherical nanoindentation, we show that nucleophile identity and polymer concentration serve as independent handles for tuning hydrogel stiffness across physiologically relevant ranges. In addition, we further demonstrate that the strategy extends beyond synthetic PEG systems by showing that hydrogels formed from PEGSNO and maleimide‐modified cold water fish skin gelatin (GelfMAL) using STS as the reaction trigger effectively support fibroblast encapsulation and display formulation‐dependent cell response in 3D cell culture. This work establishes RSNO chemistry as a previously unexplored strategy for controlled thiol‐maleimide crosslinking, enabling reproducible formation of homogeneous hydrogel networks under mild, cytocompatible conditions. The inherent NO‐releasing capacity of RSNO‐modified polymers further suggests potential for dual‐function materials in which network formation and local NO delivery are chemically coupled.

## 2 Results and Discussion

### 2.1 Characterization of PEG‐Nitrosothiol (PEGSNO)

S‐nitrosylation of 4‐arm PEG‐thiol (PEGSH) MW 10 kDa was performed with sodium nitrite under acidic conditions (pH <3) (Figure 1A). The reaction showed a near‐quantitative degree of modification, as determined by quantifying free thiols before and after nitrosylation via Ellman's assay (Figure S1, Table S1). UV/Vis spectroscopy of PEGSNO showed a strong absorbance peak at 330 nm and a weaker peak at 545 nm, both characteristic for primary aliphatic RSNOs (Figure 1B) [21]. In the ^1^H‐NMR spectrum of PEGSNO, the alpha‐methylene resonance of PEGSH at ∼2.77 ppm was absent, which is consistent with near‐quantitative end‐group modification. Moreover, the alpha‐methylene resonance of primary aliphatic RSNOs is reported to appear in the 3.9‐3.5 ppm region [22], thus overlapping with the PEG‐backbone methylene signal of PEGSNO in the same region of the spectrum (Figure 1C). Lastly, the ^15^N‐NMR spectrum of PEGS^15^NO showed a resonance at 769.55 ppm, in agreement with primary aliphatic RSNOs (Figure 1D) [23].

**FIGURE 1:**
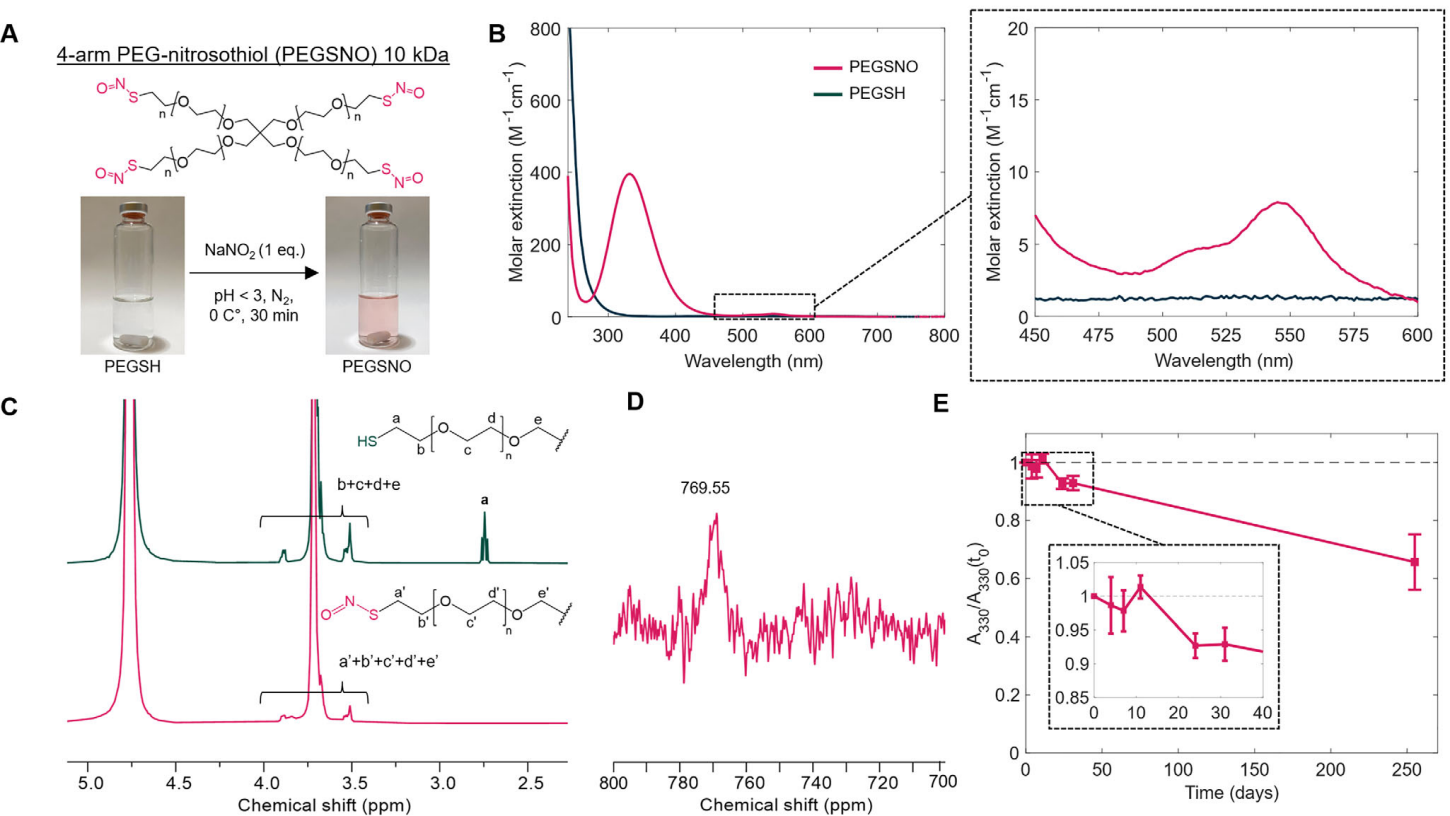
Synthesis and characterization of PEG‐nitrosothiol. (A) S‐nitrosylation of PEG‐thiol was carried out under acidic conditions using sodium nitrite, yielding nearly quantitative conversion to PEGSNO. (B) UV/Vis absorbance spectra showing molar extinction coefficients for PEGSH (dark green) and PEGSNO (pink), highlighting the characteristic S─NO peak around 545 nm for PEGSNO. (C) ^1^H‐NMR spectra of PEGSH and PEGSNO, where shift of the α‐methylene signal confirmed successful end‐group modification. (D) ^15^N‐NMR spectrum of PEGS^15^NO showed a single peak centered at 769.55 ppm. (E) Stability of PEGSNO in solution over eight months measured as the change in the absorbance peak at 330 nm via UV/Vis spectroscopy.

To evaluate the stability of the RSNO groups in PEGSNO over time, we prepared stock solutions of PEGSNO at 7% w/v in Dulbecco's Phosphate Buffered Saline (DPBS), stored them at 4°C protected from light, and checked their UV/Vis spectrum at different time points. During the first 30 days, there was a <8% decrease in the absorbance peak at 330 nm, while after >eight months in solution there was only a ∼30% decrease in the absorbance peak at 330 nm (Figure 1E), with no noticeable turbidity or gelation of the stock solution. This long‐term stability is remarkable not only relative to the previously reported RSNO compounds [21, 24] and RSNO‐modified polymers [18, 19], but also compared to that of thiol‐modified 4‐arm PEG, which oxidized overnight even when stored in DPBS at 4°C protected from light. We attribute this enhanced solution stability, at least in part, to the protective microenvironment of the PEG matrix, which is known to slow small‐molecule RSNO decomposition by restricting radical diffusion and favoring geminate recombination of the NO and thiyl radicals back to the nitrosothiol [25, 26]. At the concentration used here (7% w/v), PEG self‐association into hydrated clusters [27, 28] would further shield a fraction of the RSNO groups from the main decomposition triggers (light, oxygen, and trace metals). A detailed mechanistic study is beyond the scope of this work, but this interpretation is consistent with the higher solution stability reported for other polymer‐bound RSNOs relative to small molecules [18] and with the reduced solvent accessibility that stabilizes buried protein‐bound nitrosothiols [29]. Conversely, the lyophilized PEGSNO was found to be sensitive to air and light, as the product lost its characteristic pink color and turned water‐insoluble after approximately 10 min of air exposure. Storage of the lyophilized PEGSNO under vacuum and protected from light at −20°C proved to keep the product stable until solubilization for at least six months. It is important to note that the vacuum atmosphere resulted from sealing the vials with lyophilization stoppers at the end of the freeze‐drying cycle, and that storage under an inert atmosphere like argon is an equally suitable alternative for excluding air and moisture.

### 2.2 Nucleophile‐Induced Decomposition of PEGSNO

The main non‐enzymatic pathways by which RSNOs undergo decomposition are (i) direct photolysis, (ii) reduction catalyzed by metal ions, or (iii) nucleophilic attack to the N atom of the S─N bond leading to a transnitrosation (i.e., transfer of the NO group to a nucleophile) [21, 24, 30]. Of these, only nucleophilic attack at the nitroso nitrogen results in the direct regeneration of the parent thiol, while transferring the nitroso group to a recipient nucleophile [16, 31]. In contrast, direct photolysis and metal‐catalyzed reduction proceed via homolytic cleavage of the S─N bond, yielding thiyl radicals (RS•) that later dimerize into disulfides [24]. Thus, we hypothesized that using a suitable nucleophile would allow us to control the decomposition of RSNO groups in PEGSNO. This decomposition would lead to gradual, in situ regeneration of thiols that could participate in network‐forming reactions with a maleimide‐modified counterpart within the same reaction pot, thus overcoming kinetic constraints that prevent homogeneous mixing prior to network formation. This would result in the formation of homogeneous hydrogels via the step‐growth thiol‐maleimide Michael‐type addition (Figure 2A). A suitable nucleophile for this system must not be cytotoxic at concentrations in the order of tens of mM (slight to moderate molar excess relative to RSNOs), and must not permanently disable maleimides, which would impede the network‐forming reaction of the hydrogel system. Based on these criteria we decided to test SA and STS side‐by‐side as potential decomposition agents (Figure 2B), since their reactivity toward small‐molecule RSNOs has been previously reported [32, 33].

**FIGURE 2:**
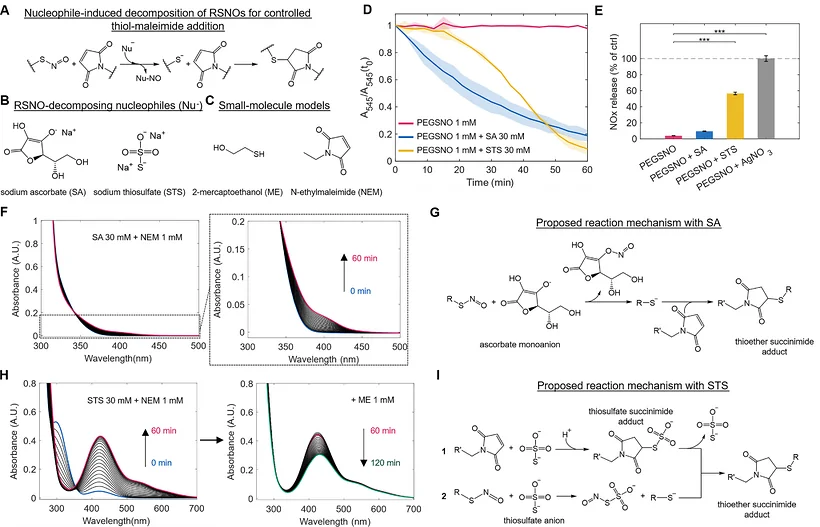
Reaction pathways and kinetics of PEGSNO decomposition by SA and STS. (A) Schematic of the nucleophile‐induced decomposition of RSNOs enabling controlled thiol‐maleimide addition: Nucleophilic attack on the RSNO releases the free thiol, which undergoes Michael‐type addition to the maleimide to form a stable thioether succinimide adducts. (B) Chemical structures of the nucleophiles selected for triggering RSNO deprotection and thiol‐maleimide addition. (C) Chemical structures of the small molecule thiol (2‐mercaptoethanol, ME) and maleimide (N‐ethylmaleimide, NEM) used as low‐molecular weight models for mechanistic studies. (D) Time‐dependent UV/Vis monitoring of PEGSNO decomposition (normalized absorbance at 545 nm, 0–60 min) in the presence of SA (blue) or STS (yellow), compared to spontaneous decay (pink). Both nucleophiles accelerate PEGSNO decomposition relative to the untreated control. (E) Quantification of NOx release following nucleophile‐induced PEGSNO decomposition by Griess assay, expressed as percentage of silver nitrate‐induced decomposition (PEGSNO + AgNO_3_). The near‐zero signal observed with SA is attributable to interference of excess ascorbate with the Griess colorimetric reaction rather than an absence of NOx release, consistent with the decomposition kinetics shown in (D). (F) UV/Vis absorbance spectra of SA with NEM over 60 min. No distinct new absorption band is observed, yet ^1^H‐NMR, TLC, and LC‐MS (Figure S6) confirm NEM consumption, indicating formation of a weakly UV‐active or spectrally overlapping product. (G) Proposed mechanism of the SA‐mediated decomposition of RSNOs for the formation of thioether bonds between thiols and maleimides. (H) UV/Vis absorbance spectra recorded over 60 min for STS mixed with NEM, showing the emergence of a new absorbance band consistent with formation of a thiosulfate succinimide adduct (left); subsequent addition of ME (1 mM) leads to decay of this intermediate absorbance peak over 120 min, consistent with thiol‐mediated displacement and thioether bond formation (right). (I) Proposed mechanism of the STS‐mediated decomposition of RSNOs for the formation of thioether bonds between thiols and maleimides.

We first investigated the kinetics of PEGSNO decomposition at 37°C and in the presence of a large molar excess (30x) of SA or STS by measuring the decay of the absorbance peak at 545 nm (Figure 2D). To prevent the decomposition of RSNOs by alternative pathways mediated by light or metal ions, the reactions were carried out protected from light and in the presence of EDTA 0.1 mM. Both SA and STS showed a clear decay of the 545 nm peak over 1 h while the control sample of PEGSNO alone displayed no significant change (Figure 2D), confirming that the decomposition of the RSNOs in PEGSNO was triggered only by the addition of the nucleophiles and was not heat‐induced. Specifically, the decomposition of PEGSNO with SA followed pseudo‐first‐order kinetics, as reported previously for small‐molecule RSNOs when using a large SA excess and in the absence of transition metal ions like Cu^2+^, with a reaction rate *k* of 4.68 × 10^−4^ ± 5.73 × 10^−5^ s^−1^ and an estimated half‐life of 1.50 × 10^3^ ± 1.87 × 10^2^ s (Figure S2, Table S2). This was, however, not the case for STS, as the reaction did not follow pseudo‐first‐order nor second‐order kinetics, suggesting a complex reaction mechanism. The estimated empirical half‐life for this reaction was 2.48 × 10^3^ ± 3.51 × 10^1^ s (Figure S3). To further confirm S─N bond cleavage, we assessed PEGSNO decomposition induced by STS or SA (30 mM, 1 h, 37°C; 1 mM RSNO) using two complementary approaches: Griess assay to detect released nitric oxide (NO)‐related species (NOx), and Ellman's assay to quantify the regenerated free thiols. In the Griess assay, (Figure 2E), STS effectively triggered NOx release (>60% relative to the positive control with silver nitrate), consistent with S─N bond cleavage and release of the nitroso group into solution. In contrast, the SA condition yielded a near‐zero signal, which was attributed to chemical interference by excess ascorbate with the Griess colorimetric reaction. A control experiment in which 1 mM sodium nitrite was added to the SA reaction mixture confirmed the suppression of the Griess signal, consistent with ascorbate‐mediated reduction of nitrite and/or quenching of the diazonium intermediate prior to color development. For Ellman's assay, samples were purified by centrifugal ultrafiltration (3 kDa MWCO) prior to measurement to remove excess nucleophiles, which interfere with 5,5‐dithio‐bis‐(2‐nitrobenzoic acid) (DTNB)‐based thiol quantification. However, the expected thiol signal was not recovered in either condition (Figure S4). This was attributed to secondary reactions of the regenerated thiols with the large molar excess of nucleophiles. In the case of STS, free thiols are likely to be converted to Bunte salts through reaction with excess thiosulfate or its oxidation products. In the SA condition, the partial signal may reflect incomplete removal of ascorbate, which reduces DTNB independently. Taken together, these results confirm that both SA and STS trigger S─N bond cleavage in PEGSNO under mild conditions, though through different mechanisms and at different rates.

Next, we investigated the interaction of the two nucleophiles at a large excess (30x) with maleimide groups using N‐ethylmaleimide (NEM) as a model maleimide (Figure 2C). UV/Vis kinetic analysis of SA with NEM (Figure 2F) showed a spectral evolution qualitatively similar to that of SA alone (Figure S5), with a gradual increase in the long‐wavelength tail consistent with SA oxidation products [34]. While these spectra did not reveal a distinct new absorption band attributable to an SA‐maleimide adduct, subsequent analyses showed consumption of NEM in the presence of SA: In ^1^H‐NMR (3 eq. SA) the characteristic maleimide vinylic signal at ∼6.9 ppm disappeared after a 1 h incubation (Figure S6), and LC‐MS analysis of a sample containing NEM 1 mM and SA 30 mM showed a UV peak at 1.21 min, the mass spectrum of which contained a principal ion at *m/z* 302, consistent with an ascorbate‐maleimide Michael adduct (Figure S7). Together, these data suggest conversion of NEM into a weakly UV‐active and/or spectrally overlapping product, consistent with a slow nucleophile‐ or redox‐enabled transformation of the maleimide under these conditions. Importantly, the thiol‐maleimide Michael‐type addition is intrinsically fast, with second order rate constants for small molecules on the order of ∼10^2^–10^3^ M^−1^s^−1^ at physiological pH [35, 36]. Even at the 1 mM maleimide used here the pseudo‐first‐order capture rate exceeds the SA‐triggered PEGSNO decomposition (*k* = 4.68 × 10^−4^ ± 5.73 × 10^−5^ s^−1^; Figures 2D and S2) by two to three orders of magnitude. Regenerated thiols are therefore consumed by maleimides essentially as fast as they are liberated, so thiol‐maleimide coupling is kinetically favored over the direct SA‐maleimide reaction despite the large excess of SA. This margin is conservative for the slower polymer‐bound reaction and is expected to widen further in the hydrogel system. Accordingly, a mechanism is proposed in which SA primarily triggers thiol regeneration from PEGSNO, followed by network formation through step‐growth thiol‐maleimide Michael‐type addition, with a minor competing SA‐maleimide side reaction (Figure 2G). Conversely, STS showed a clear interaction with NEM, as the UV/Vis kinetic analysis evidenced a fast decay of the band at 300 nm corresponding to NEM (Figure 2H) and in ^1^H‐NMR the characteristic vinylic signal of NEM at ∼6.9 ppm was absent after a 1 h incubation (Figure S8). Moreover, the UV/Vis spectra showed a growing peak centered at 425 nm, which was attributed to the formation of a conjugated thiosulfate succinimide Michael adduct. Interestingly, the peak decayed over time upon the addition of 2‐mercaptoethanol (ME) equimolar to NEM, which evidenced that the conjugated adduct is thiol‐labile, yet the NEM peak at 300 nm was not recovered. Notably, the kinetics of both adduct formation and thiol‐mediated displacement were buffer‐dependent, with the fastest rates observed in sodium phosphate 100 mM (Figure S9), suggesting a role of ionic strength or specific ion effects on the nucleophilic substitution steps. For subsequent hydrogel experiments, HEPES 25 mM + EDTA 0.1 mM (to prevent metal ion‐mediated RSNO decomposition pathways) pH 7.4 was selected as a compromise between reaction kinetics and cell compatibility. Taken together, these data suggest a scenario where thiols displace STS from the thiosulfate succinimide adduct, most likely via direct nucleophilic substitution or retro‐Michael addition (Figure 2I).

### 2.3 Nucleophile‐Mediated Crosslinking of Homogeneous Hydrogels via Thiol‐Maleimide Michael‐Type Addition in a PEG‐Based Polymer System

We then proceeded to study the hydrogel crosslinking in a fully defined system with SA or STS as nucleophiles, PEGSNO as a source of functional thiols, and maleimide‐modified PEG (PEGMAL) of the same architecture and size as PEGSNO (4‐arm, MW 10 kDa) (Figure 3A). To first explore the lower limit of the hydrogel crosslinking window as a function of nucleophile type, nucleophile concentration, and hydrogel precursor concentration (1:1, RSNO:maleimide), we screened 40 different compositions for the formation of stable hydrogels (Figure 3B). Three sets of controls were included: hydrogels prepared with PEGSH instead of PEGSNO across the same polymer content range, PEGMAL + nucleophile alone, and PEGSNO + nucleophile alone (Table S3). The experiment was done by dispensing combinations of stock solutions of the precursors to a final hydrogel volume of 3 µL on a droplet microarray (DMA) slide, which consisted of 80 hydrophilic round spots (Φ 3.2 mm) surrounded by superhydrophobic borders on the surface of a 75.6 × 25.0 × 1.0 mm borosilicate glass slide [37, 38]. The slides with dispensed samples were incubated at 37°C for 30 min, washed by five subsequent immersions in DPBS to remove the samples that did not form a gel, and left overnight in a solution of rhodamine 6G 10 µM in DPBS at room‐temperature to allow swelling to equilibrium (Figure 3B). The incubation time was selected as a design constraint to preserve the usability of the new system and to avoid unwanted effects of longer incubation periods‐like cell precipitation or a compromise in their viability due to lack of nutrients. Fluorescence images of the slides showed that 50 mM was the minimum nucleophile concentration that induced stable hydrogel formation in formulations with PEGSNO, and that STS triggered this process starting at 3% w/v total polymer content (i.e., PEGMAL 1.5% w/v + PEGSNO 1.5% w/v), while SA did so starting at 3.5% w/v (Figure 3C). Controls consisting of PEGMAL 2% w/v + STS 50 mM and PEGMAL 2% w/v + SA 50 mM failed to form gels.

**FIGURE 3:**
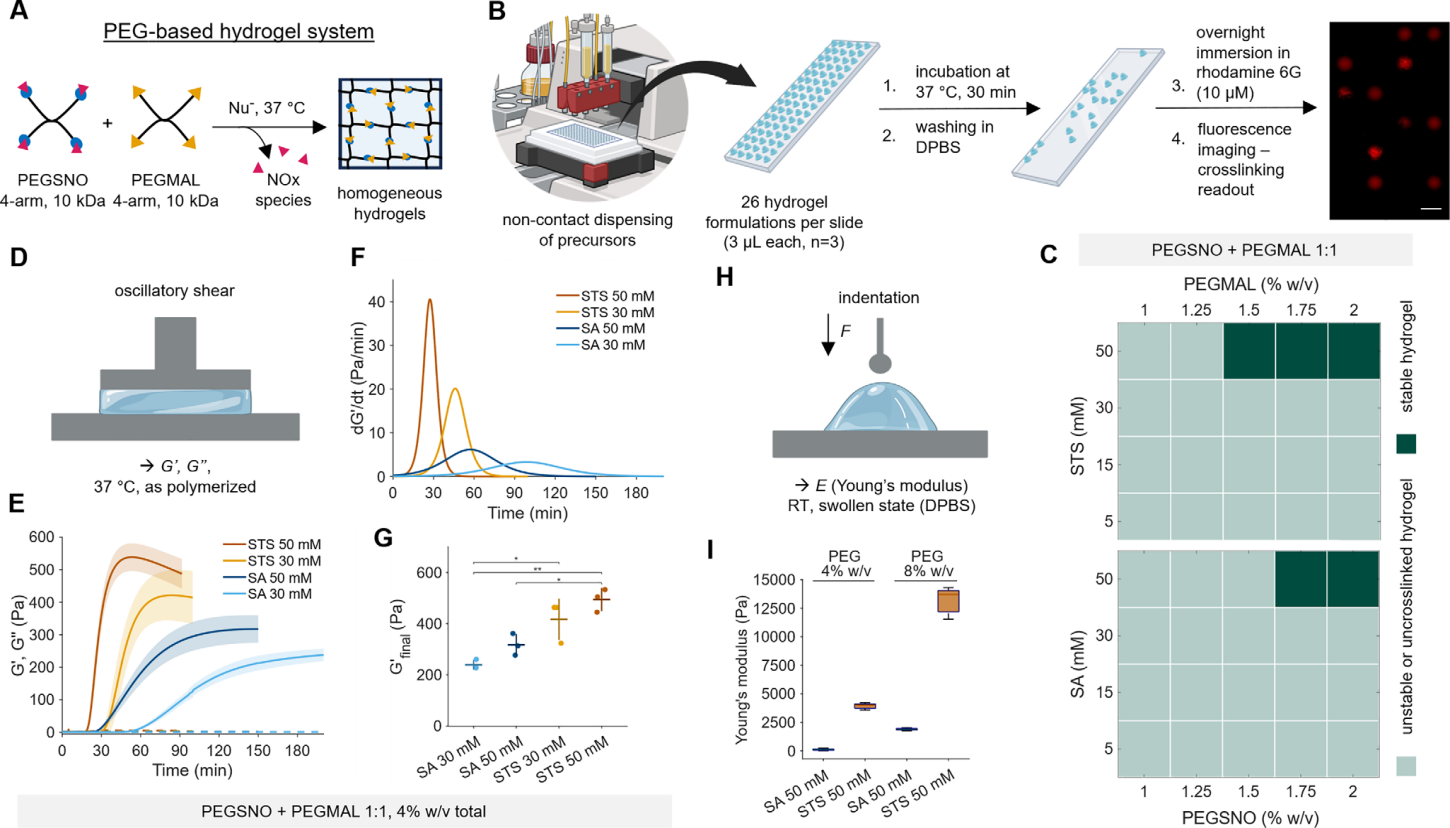
Hydrogel formations from PEG‐SNO–modified biomaterials upon treatment with SA or STS. (A) Schematic of the hydrogel crosslinking mechanism: PEGSNO serves as a masked thiol precursor, that is activated upon nucleophilic attack at the nitroso nitrogen, releasing NO‐related species and regenerating free thiols that undergo Michael addition with pendant maleimide groups. (B) High‐throughput crosslinking screen using the DMA platform, where 46 formulations and controls (3 µL each, *n* = 3) were dispensed onto hydrophilic spots, incubated at 37°C for 30 min, washed in DPBS, and swollen overnight in rhodamine 6G. Fluorescence intensity served as binary crosslinking readout. (C) Stable hydrogel formation window of the system at total PEG (PEGMAL + PEGSNO, 1:1) concentrations between 2% and 4% w/v total polymer content with STS (top) and SA (bottom) ranging between 5 and 50 mM. A 30 min gelation window at 37°C was imposed as a design constraint for usability. (D, E, F) Oscillatory shear rheology of PEGSNO‐PEGMAL 1:1 at 4% w/v total at 37°C. Storage (G‵) and loss (G‵‵) moduli kinetics (E) and gelation rate dG‵ dt^−1^ (F) showed nucleophile‐ and concentration‐dependent crosslinking for STS and SA at 30 and 50 mM. (G) G‵ _final_ calculated at the end of gelation kinetics experiments showed that STS produced stiffer gels than SA at the same polymer concentration (*p < 0.05, **p < 0.01). (H) The mechanical properties of the hydrogels in the swollen state (DPBS, room temperature) were measured via spherical indentation. (I) Young's modulus of 4% and 8% w/v PEG hydrogels showed a strong concentration dependence and confirmed the higher stiffness of STS‐crosslinked hydrogels. Scale bar: (B) 3 mm. Schemes were created with BioRender.com.

We then proceeded to study the gelation kinetics of the system using oscillatory rheology in HEPES 25 mM + EDTA 0.1 mM pH 7.4 buffer, fixing the total polymer concentration to 4% w/v while varying the nucleophile type (SA or STS) and concentration (30 or 50 mM) (Figure 3D and E). Results showed the formation of predominantly elastic networks (loss modulus G″ < 5% of the storage modulus G′) [39] for all formulations, which provided additional evidence of the network structures being governed by covalent thiol‐maleimide bonding. Gelation curves were fitted to a 4‐parameter logistic model (Table S4) to extract the time derivative of the storage modulus (dG‵ dt^−1^), while the storage modulus at the end of the gelation kinetics measurements (G‵_final_) was determined directly from the kinetics experiments. The derivative plots revealed that STS‐crosslinked systems exhibited faster and more temporally concentrated gelation events, with sharp dG‵ dt^−1^ peaks reaching to ∼40 Pa min^−1^ for STS 50 mM at approximately 35 min, compared to the broader, lower‐amplitude peaks observed for SA (∼6 Pa min^−1^ maximum for SA 50 mM) (Figure 3F). This indicated that thiol‐maleimide crosslinking mediated by STS proceeded with faster reaction kinetics than with SA under these conditions, resulting in a higher instantaneous crosslink density during network formation. Moreover, STS‐crosslinked hydrogels reached substantially higher G′_final_ values than their SA counterparts at equivalent nucleophile concentrations, with STS 50 mM yielding the stiffest networks (494 ± 45 Pa) and SA 30 mM the most compliant (239 ± 19 Pa) (Figure 3G). These differences were statistically significant between the SA and STS groups (*p* < 0.05 and *p* < 0.01 for 30 and 50 mM comparisons, respectively), while no significant difference was observed between nucleophile concentrations within each group, suggesting that nucleophile type is a stronger determinant of final network stiffness than nucleophile concentration under the conditions tested. Interestingly, the STS 50 mM formulation reproducibly showed a transient maximum in G′ followed by a modest decline, with a smaller decrease also observed for STS 30 mM. The more slowly gelling SA formulations did not reach a clear terminal maximum within the measurement window, so a comparable late‐stage response cannot be excluded. The decline coincided with continued development of tensile axial force under the fixed‐gap geometry (Figure S10). We therefore hypothesize that, once continued crosslinking contributes progressively less to the formation of elastically effective connections, contraction‐associated stress redistribution and network relaxation can produce modest softening without loss of network integrity.

Since bulk rheology of as‐polymerized hydrogels does not capture the mechanical environment experienced by embedded cells in a swollen polymeric network, spherical indentation was used to characterize the compressive stiffness of the hydrogels at swelling equilibrium (Figure 3H). Hydrogels at 4% and 8% w/v total polymer concentration (PEGMAL + PEGSNO, 1:1) were prepared with both nucleophiles at 50 mM, incubated for 30 min at 37°C, washed twice with DPBS, and subsequently incubated overnight with fresh DPBS to allow swelling until equilibrium. Consistent with the rheology experiments, STS‐crosslinked hydrogels were significantly stiffer than their SA‐crosslinked counterparts at matched polymer concentration and increasing the polymer content from 4% to 8% w/v resulted in hydrogels with a higher Young's modulus regardless of the nucleophile type (Figure 3I). All four formulations were statistically distinct from one another, demonstrating that both polymer concentration and nucleophile type can be used as independent handles to tune hydrogel stiffness across a physiologically relevant range. The chemical bonding underlying these networks was studied directly during the gelation process by Raman spectroscopy (785 nm, confocal) on the 8% w/v (total polymer content, 4% w/v PEGMAL + 4% w/v PEGSNO) PEG‐based system with STS 50 mM (Figure S11). The progressive attenuation of the maleimide‐associated bands (C═C stretch at 1588 cm^−^^1^ and imide C═O stretch at 1773 cm^−^^1^) is consistent with maleimide consumption through thiol‐maleimide Michael‐type addition during network formation, further supporting the covalent basis inferred from the low G′′/G′ ratios (Figure 3E).

As mentioned before, the structural heterogeneity of thiol‐maleimide hydrogels caused by the gelation speed outpacing the mixing of the precursors is the main limitation for their practical use. To assess the homogeneity of the hydrogels formed via nucleophile‐triggered polymerization, we analyzed fluorescence images of the hydrogels formed with 4% w/v total PEG and 50 mM of SA or STS, using PEGSH‐based hydrogels as heterogeneous controls (Figure 4A). Note that these were the same hydrogels prepared by non‐contact dispensing of the precursors on the DMA platform (Figure 3B and C), since manual mixing or pipetting is not feasible for PEGSH hydrogels due to the extremely fast crosslinking clogging the pipette tips. Normalized fluorescence intensity profiles measured along the hydrogel diameter showed high variability in the PEGSH control, whereas both PEGSNO samples showed smooth, uniform profiles consistent with homogeneous polymer network distribution due to much slower reaction kinetics (Figure 4B). Additionally, two dimensionless parameters were used to quantify intensity non‐uniformity within the hydrogels (coefficient of variation of the fluorescence intensity, Figure 4C) and shape irregularity at their boundaries (edge heterogeneity index, Figure 4D). Both parameters were significantly higher in the PEGSH controls than in either PEGSNO condition, confirming that nucleophile‐triggered regeneration of thiols from PEGSNO improved hydrogel homogeneity regardless of the nucleophile used. This decoupling of mixing from gelation is not only responsible for the improved homogeneity observed here, but should also make it possible to use the system for casting larger, homogeneous bulk gels, in contrast to the near‐instantaneous crosslinking that limits the scale‐up of conventional thiol–maleimide hydrogels. Notably, hydrogels formed with SA 50 mM appeared larger in area and had less well‐defined boundaries than those formed with STS or the PEGSH control, consistent with higher swelling and a lower crosslinking density, in agreement with the indentation data in the same swollen state (Figure 3I).

**FIGURE 4:**
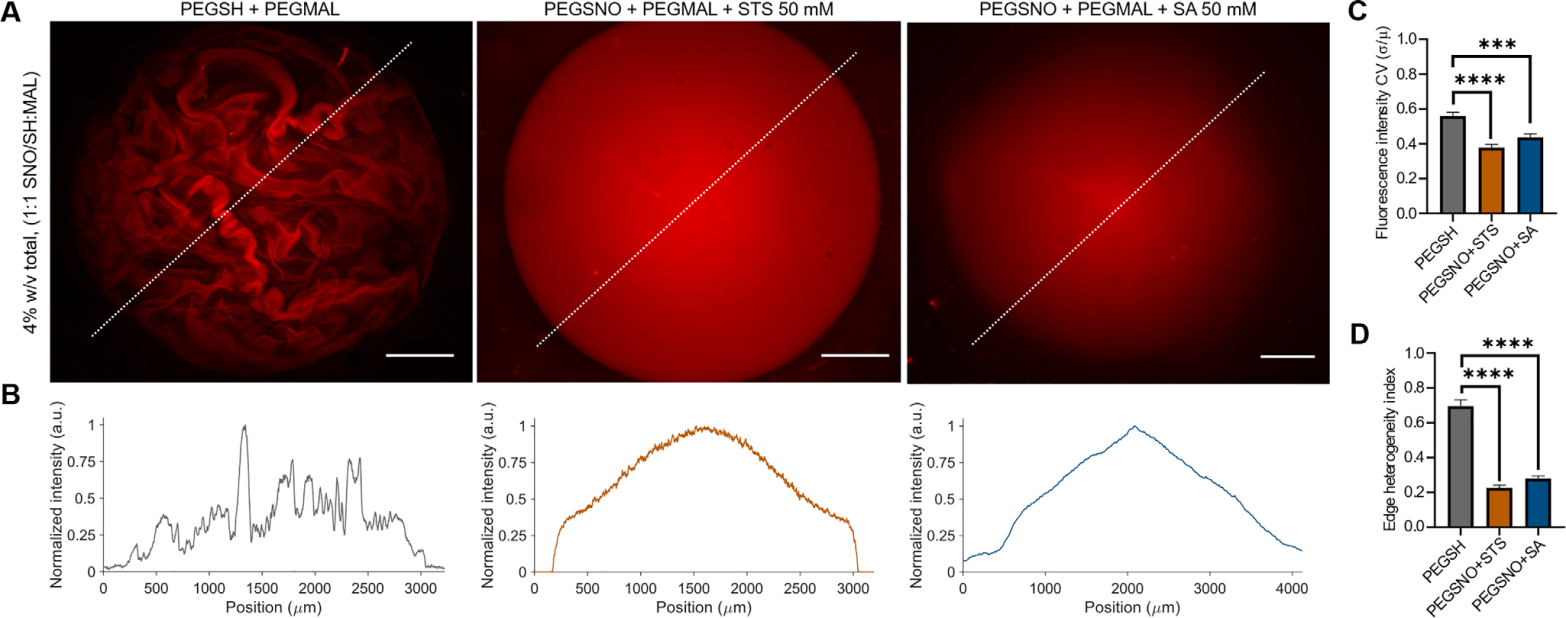
(A) Fluorescence images of hydrogels (4% w/v total, 1:1 SNO/SH:MAL ratio) swollen overnight in a rhodamine 6G solution, comparing a PEGSH control with PEGSNO formulations where polymerization was triggered by STS (50 mM) or SA (50 mM). Dashed lines indicate the position of the intensity profiles shown in (B). (B) Normalized fluorescence intensity profiles across the gel diameter, showing spatially uniform dye distribution in PEGSNO‐based hydrogels (middle and right) and heterogeneous distribution in the PEGSH control (left). (C) Coefficient of variation (CV) of intra‐hydrogel fluorescence intensity and (D) Sobel‐based edge heterogeneity index confirmed that PEGSNO‐based hydrogels exhibited significantly lower structural heterogeneity than the PEGSH control for both nucleophiles (***p < 0.001, ****p < 0.0001). Scale bar: (A) 500 µm.

### 2.4 PEGSNO‐Enabled Hydrogels Support 3D Cell Encapsulation With Formulation‐ and Cell‐Type‐Dependent Cytocompatibility

After establishing that STS and SA could effectively trigger hydrogel crosslinking in a PEG‐based thiol‐maleimide system, we next asked whether the system could be translated into a 3D cell culture platform using a bioactive, cell‐adhesive biopolymer, since PEG lacks cell‐binding motifs. We synthesized maleimide‐functionalized cold water fish skin gelatin (GelfMAL) and determined its degree of modification using ^1^H‐NMR spectroscopy (Figure S12). For this translation to be successful, the nucleophile must be cytocompatible at the concentrations required to trigger hydrogel crosslinking, and the crosslinking process itself must support cell viability across a relevant range of matrix stiffnesses. We addressed this using NIH/3T3 immortalized mouse embryonic fibroblasts as a widely used model for 3D hydrogel studies and for fibroblast responses to reactive nitrogen species (RNS) in vitro.

We first evaluated the cytotoxicity of the nucleophiles across a concentration range relevant to hydrogel crosslinking (0.9‐100 mM) on NIH/3T3 cells by studying their metabolic activity and membrane integrity after 30 and 120 min exposures (time points chosen to mimic the gelation window and the early post‐gelation period, respectively). Resazurin‐based metabolic activity measurements (Figure 5A) showed that SA elicited a dose‐dependent drop in metabolic activity above ∼3.75 mM, with near‐complete loss of activity at concentrations relevant for triggering hydrogel crosslinking (≥30 mM). The LDH‐release assay (Figure 5B) showed a similar trend, where concentrations ≥ 30 mM produced LDH release equivalent to ∼50% of the lysis‐positive control. Taken together, these data ruled out SA as a viable nucleophile for cell encapsulation studies with NIH/3T3 cells. While SA elicited cytotoxic responses in both assays, consistent with its reported cytotoxicity at sub‐millimolar concentrations via generation of hydrogen peroxide in standard cell culture medium [40, 41], STS showed no significant reduction in cell metabolic activity at concentrations up to 50 mM, and membrane integrity was preserved under the same conditions. The results are consistent with reports showing that STS is well tolerated by mammalian cells at millimolar concentrations and with its clinical use in gram‐scale doses as a chemo protectant against cisplatin‐induced toxicity [42] and as an antidote for cyanide poisoning [43]. On this basis, we selected STS at concentrations up to 50 mM as the cytocompatible trigger for NIH/3T3 cell encapsulation.

**FIGURE 5:**
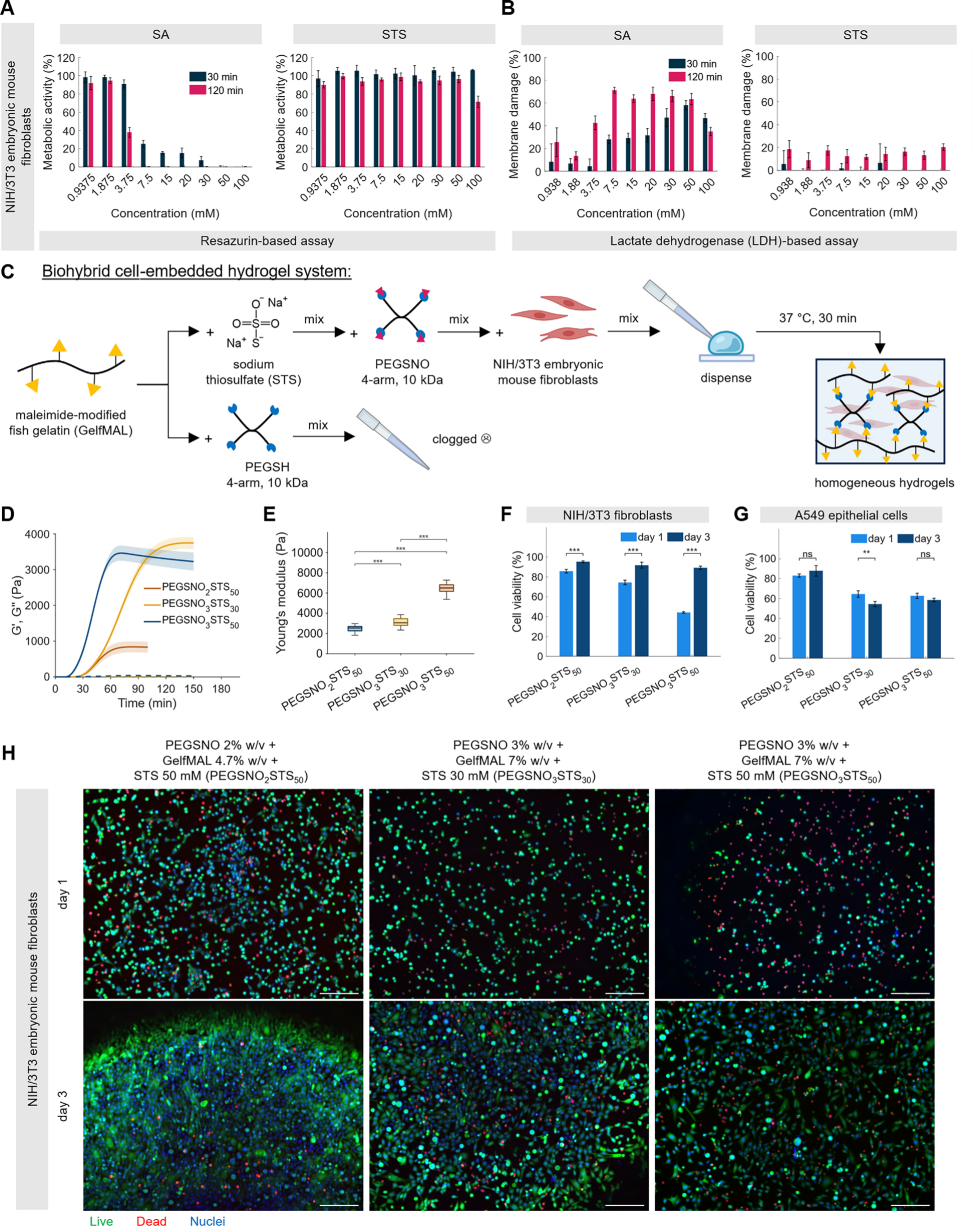
PEGSNO/GelfMAL hydrogels support fibroblast and epithelial cell encapsulation and enable stiffness tuning via polymer content and STS concentration. (A) Metabolic activity of NIH/3T3 cells in monolayer measured by a resazurin reduction assay after 30 and 120 min of exposure to SA (left panel) or STS (right panel) across concentrations spanning 0.9‐100 mM. (B) LDH release from NIH/3T3 cells under the same exposure conditions as in (A), expressed as membrane damage relative to the lysis‐positive control. (C) Schematic of the cell encapsulation workflow: PEGSNO, GelfMAL, and STS are mixed and used to resuspend cells. The cell‐embedded precursor is then dispensed into well plates, where gelation precedes at 37°C over 30 min. This step‐by‐step approach is not feasible with PEGSH, since the fast gelation results in clogging of the pipette tip when attempting to mix the precursors. (D) Oscillatory rheology gelation kinetics at 37°C of PEGSNO_2_STS_50_, PEGSNO_3_STS_30,_ and PEGSNO_3_STS_50_ (compositions indicated on top of the microscopy images, Panel H), showing the storage modulus (G‵, solid lines) and loss modulus (G‵‵, dashed lines) over time. (E) Young's moduli of PEGSNO_2_STS_50_, PEGSNO_3_STS_30_ and PEGSNO_3_STS_50_ gelled by incubation at 37°C for 30 min, and measured in the swollen state by spherical indentation. (F,G) Cell viability at days 1 and 3 across formulations, quantified from live/dead staining, for NIH/3T3 fibroblasts (F) and A549 human lung carcinoma epithelial cells (G). (H) Representative fluorescence images of NIH/3T3 fibroblasts encapsulated in PEGSNO_2_STS_50_, PEGSNO_3_STS_30,_ and PEGSNO_3_STS_50_ stained with calcein‐AM (green, live cells), propidium iodide (red, dead cells), and Hoechst (blue, nuclei) at day 1 and 3 post‐encapsulation. Cells double stained with calcein‐AM and propidium iodide were counted as dead. Scale bars: 200 µm. Data in (A), (B), (F) and (G) are shown as mean ± SD, *n* = 4. Statistical analysis of viability data was performed using two‐way ANOVA (factors: gel formulation and timepoint) with Tukey‐Kramer post‐hoc tests (ns *p* > 0.05, **p < 0.01, ***p <0.001). Schemes were created with BioRender.com.

Next, we encapsulated NIH/3T3 fibroblasts in three different hydrogel formulations chosen to decouple contributions of polymer content and STS concentration, while preserving the 1:1 RSNO:MAL ratio used for studying the PEG‐based system: PEGSNO 2% w/v + GelfMAL 4.7% w/v + STS 50 mM (PEGSNO_2_STS_50_); PEGSNO 3% w/v + GelfMAL 7% w/v + STS 30 mM (PEGSNO_3_STS_30_); and PEGSNO 3% w/v + GelfMAL 7% w/v + STS 50 mM (PEGSNO_3_STS_50_). Oscillatory rheology of the three formulations at 37°C (Figures 5D, S13) showed that PEGSNO_3_STS_50_ gelled fastest and to the highest plateau modulus, followed by PEGSNO_3_STS_30_, while PEGSNO_2_STS_50_ formed a markedly weaker network. For cell experiments, STS was first mixed with the polymer precursors, and the resulting mixture was used to resuspend a cell pellet by gentle pipetting. 8 µL of the cell‐embedded precursor mixture were pipetted into four well plates and incubated inverted at 37°C in a CO_2_ incubator for 30 min to prevent cell sedimentation and allow gelation (Figure 5C). The formed cell‐embedded gels were then washed twice with DPBS and maintained in DMEM supplemented with 10% FBS for one or three days before analysis. Live/dead staining at day 1 showed cell viabilities of approximately 80% in PEGSNO_2_STS_50_ and PEGSNO_3_STS_30_ (Figure 5F,H). PEGSNO_3_STS_50_, which was the formulation that combined the highest STS concentration with the highest PEGSNO content, showed a marked population of PI‐positive cells at day 1, with viability dropping to ∼45% (Figure 5F,H). By day 3, live/dead viability had risen to ≥90% across all formulations for NIH/3T3 fibroblasts. This increase suggested a combined effect of survival and proliferation of the fibroblasts over the culture period window, while the reduced viability at day 1 in PEGSNO_3_STS_50_ reflected an acute stress imposed during gelation that the cell population subsequently outgrew, rather than a sustained cytotoxic effect of the matrix, and that all three formulations support NIH/3T3 survival over the timescale relevant for 3D culture.

To test whether this acute sensitivity was specific to fibroblasts or a general property of the system, we encapsulated A549 human alveolar epithelial cells in the same three formulations (Figure 5G). At day 1, A549 cells showed the same formulation ranking as fibroblasts, with viability decreasing as PEGSNO content and STS concentration increased. In contrast to fibroblasts, A549 viability did not increase from day 1 to 3, improving only in the mildest formulation (PEGSNO_2_STS_50_) and remaining reduced in PEGSNO_3_STS_30_ and PEGSNO_3_STS_50_. Since STS was well tolerated by A549 cells in monolayer (Figure S14), this indicated that the acute response scaled with the RNS‐generating capacity of the formulation and is tolerated in a cell‐type‐dependent manner rather than being an intrinsic, cell‐independent property of the system. In addition, depth imaging of the A549‐embedded gels at day 3 confirmed that the 3% PEGSNO formulations distributed cells throughout the gel volume (Movies S2 and S3), whereas in the slower‐forming PEGSNO_2_STS_50_ formulation a fraction of cells settled toward the gel‐air interface before the weaker network set (Movie S1, Figure S14), consistent with its lower gelation rate (Figure 5D). For slower‐gelling formulations, performing intermediate inversions of the plates with the gels throughout the incubation period should aid in achieving a true 3D distribution of cells.

The acute cytotoxicity observed in PEGSNO_3_STS_50_ is consistent with a dose‐dependent effect of the RNS released during PEGSNO decomposition. Under reducing conditions, RSNOs can generate a mixture of products including NO, nitroxyl (HNO/NO‐), disulfides, and nitrite, with the relative contributions depending on the reducing agent and local conditions [16, 44, 45]. The three RNS in this list (NO, nitroxyl and nitrite) all exhibit a strong dose‐dependent effect on cell proliferation and viability. Low‐to‐moderate doses of NO from S‐nitrosothiols, like S‐nitroso‐N‐acetylpenicillamine (SNAP, 0.005‐0.02 mM) and S‐nitrosoglutathione (GSNO, 0.025‐0.05 mM) have been shown to promote proliferation in BALB/c 3T3 fibroblasts [46], while higher doses of SNAP (>0.03 mM) inhibit mitogenesis and proliferation in the same cell line [47, 48]. Similar biphasic behavior has been reported for nitrite, with sub‐millimolar to millimolar doses increasing proliferation and higher doses producing dose‐dependent cytotoxicity in multiple cell types [49, 50]. Nitroxyl is similarly cytotoxic to fibroblasts in the low‐millimolar range [51] and attenuates tumor proliferation and angiogenesis [52]. In PEGSNO_3_STS_50_, which contained both the highest PEGSNO content (3% w/v polymer, ∼12 mM RSNO) and the highest STS concentration (50 mM), the resulting RNS burst during gelation likely exceeded the cytocompatibility window for NIH/3T3 fibroblasts, producing the ∼45% viability observed at day 1. In PEGSNO_2_STS_50_ and PEGSNO_3_STS_30_, the lower PEGSNO content or lower STS concentration attenuated this burst, exposing cells to a dose range that supported survival and active proliferation. Matrix stiffness may contribute additionally, since PEGSNO_3_STS_50_ is also the stiffest formulation and densely crosslinked covalent networks are known to restrict fibroblast spreading independently of chemical insults.

To assess this potential mechanical contribution, we measured the Young's moduli of the three formulations in the swollen state by spherical indentation (Figure 5E). PEGSNO_2_STS_50_ and PEGSNO_3_STS_30_ displayed Young's moduli of 2.48 ± 0.33 kPa and 3.10 ± 0.39 kPa, respectively, while PEGSNO_3_STS_50_ showed a substantially higher modulus of 6.55 ± 0.66 kPa. The ∼2‐fold difference in the Young's modulus between PEGSNO_3_STS_30_ and PEGSNO_3_STS_50_, which had the same polymer content, was attributed to the change in STS concentration alone, consistent with our earlier observation that nucleophile concentration tunes the effective crosslinking density. Overall, the range of moduli accessed across the three formulations (∼2.5–6.5 kPa) falls within values relevant for soft tissue 3D culture. Across the three formulations, matrix stiffness can be tuned by modulating either polymer content (PEGSNO_2_STS_50_ vs. PEGSNO_3_STS_50_) or trigger concentration (PEGSNO_3_STS_30_ vs. PEGSNO_3_STS_50_).

As a preliminary assessment of how these differences in chemistry and mechanical properties translated into cell behavior, one sample of each formulation was fixed at day 3 post‐encapsulation and stained for F‐actin (phalloidin), Ki‐67, and nuclei (DAPI) (Figure 6). In PEGSNO_2_STS_50_ and PEGSNO_3_STS_30_, 3T3 cells showed highly spread cell bodies with extensive F‐actin‐positive protrusions, consistent with the morphology expected of fibroblasts in mechanically permissive 3D environments. Ki‐67 staining was abundant and strongly colocalized with DAPI in both formulations, indicating active cell‐cycle progression. In contrast, cells in PEGSNO_3_STS_50_ showed minimal phalloidin signal and noticeably sparser Ki‐67 staining; with overall DAPI‐positive cell density also lower than in the other two formulations. Taken together with the ∼45% viability measured at day 1 (Figure 5F), this pattern suggests that the acute cytotoxicity during gelation reduced the viable encapsulated cell population, and that the surviving cells did not fully recover spreading or proliferative activity by day 3. Since the STS concentration influences both crosslinking density and the RNS load, the suppression of spreading and proliferation in PEGSNO_3_STS_50_ cannot be assigned to either factor alone and likely reflects their combined effect.

**FIGURE 6:**
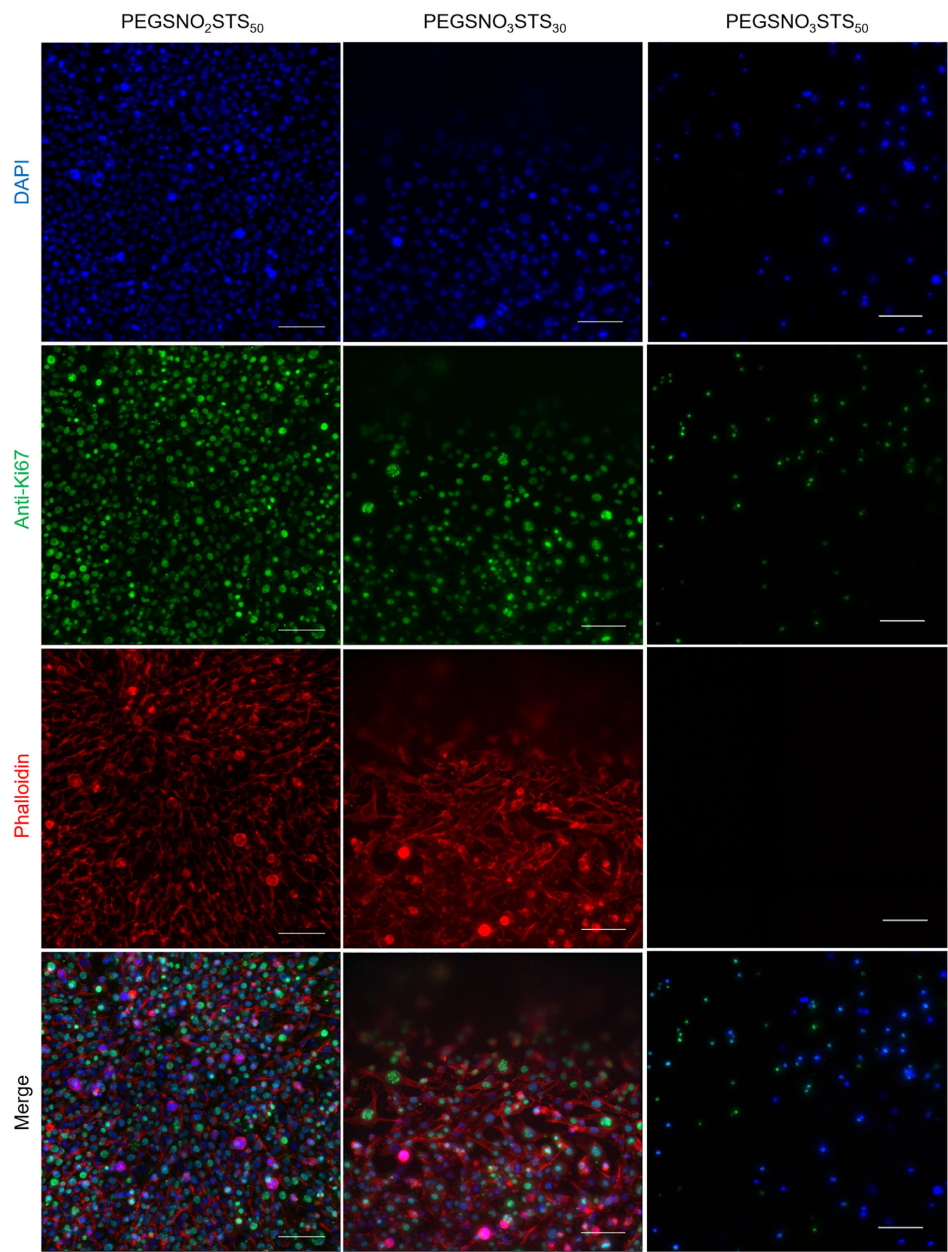
Immunostaining of NIH/3T3 fibroblasts encapsulated in PEGSNO_2_STS_50_, PEGSNO_3_STS_30,_ and PEGSNO_3_STS_50_ hydrogels at day 3 post‐encapsulation. Cells were fixed and stained for nuclei (DAPI, blue), the proliferation marker Ki‐67 (anti‐Ki‐67, green), and F‐actin (phalloidin, red); the bottom row shows the merged channels. Cells in PEGSNO_2_STS_50_ and PEGSNO_3_STS_30_ displayed spread morphologies with extensive F‐actin‐positive protrusions and abundant Ki‐67 signal colocalized with DAPI, whereas cells in PEGSNO_3_STS_50_ showed no detectable phalloidin signal, reduced Ki‐67 staining, and lower overall DAPI‐positive cell density. Scale bars: 100 µm. *n* = 1.

Overall, these data establish PEGSNO/GelfMAL as a tunable 3D culture platform in which gelation kinetics and matrix stiffness can be adjusted through polymer content and nucleophile concentration, with cytocompatibility that is formulation‐ and cell‐type‐dependent and highest in the milder formulations.

## 3 Conclusions

In this work, we introduced S‐nitrosothiols (RSNOs) as a new class of thiol‐protecting groups for controlled thiol‐maleimide crosslinking in soft hydrogels. By converting PEG‐thiols into their S‐nitroso derivatives, we suppressed premature reactivity with maleimides and enabled homogeneous mixing of polymer precursors prior to on‐demand network formation. SA and STS were evaluated side‐by‐side as nucleophilic triggers, with both effectively cleaving the S─N bond in RSNO‐modified 4‐arm polyethylene glycol (PEGSNO), but through different mechanisms and at different rates, and with distinct interactions with maleimide groups. In a fully defined PEG‐based system, both nucleophiles produced homogeneous hydrogels whose stiffness could be tuned across a physiologically relevant range (∼0.3‐13 kPa) by independently modulating the nucleophile type, nucleophile concentration or polymer content. Extending the strategy to a bioactive, cell‐adhesive system based on PEGSNO and maleimide‐modified cold water fish skin gelatin (GelfMAL) resulted in hydrogels with Young's modulus spanning ∼2.5‐6.5 kPa, tunable through polymer content or STS concentration. STS‐triggered gelation supported 3D encapsulation of NIH/3T3 fibroblasts, which reached ≥90% viability by day 3, and preliminary immunostaining indicated active spreading and cell‐cycle progression in the two softer formulations. A549 epithelial cells, by contrast, recovered only in the mildest formulation, showing that cytocompatibility in this system depends on both formulation and cell type. The stiffest formulation (PEGSNO 3% w/v + GelfMAL 7% w/v + STS 50 mM; PEGSNO_3_STS_50_), which combined the highest polymer content with the highest STS concentration and therefore the highest RNS load, marked the boundary of the chemically tolerated regime for the studied cell types. Beyond addressing a long‐standing kinetic limitation of thiol‐maleimide chemistry, this work establishes RSNOs as a new class of thiol‐protecting groups, with implications that extend beyond the hydrogel system reported here. The same protection‐activation logic could be applied to any thiol‐driven chemistry where premature reactivity, off‐target conjugation, or limited shelf‐life of thiol‐functionalized precursors is a constraint. Because S‐nitrosylation is mild, reversible, and orthogonal to most bioconjugation chemistries, RSNO‐based protection is in principle compatible with peptides, proteins, and other biomolecules, opening opportunities for storage‐stable thiol reagents that can be activated on demand under physiological conditions. In the specific context of hydrogels, the inherent capacity of RSNOs to release nitric oxide (NO) and related reactive nitrogen species (RNS), further enables a new class of dual‐function materials in which network formation and local RNS delivery are chemically coupled. This concept is relevant to applications in mechanobiology, tissue engineering, and RNS‐responsive therapeutic platforms, where matrix mechanics and locally delivered signaling species could be designed and tuned together rather than as independent variables. More broadly, we anticipate that nucleophile‐triggered denitrosation will provide a versatile chemical handle for the next generation of stimuli‐responsive materials and bioconjugation strategies.

## 4 Experimental Details

### 4.1 Materials and Chemicals

4‐arm PEG‐thiol (PEGSH, 10 kDa MW) and 4‐arm PEG‐maleimide (PEGMal, 10 kDa MW) were purchased from Xiamen Sinopeg Biotech Co., Ltd. (China). 3‐(Trimethylsilyl)propionic‐2,2,3,3‐d4 acid sodium salt (TMSP, Sigma‐Aldrich, cat. no.: 269913), deuterium oxide (Sigma‐Aldrich, cat. no.: 151882), sodium nitrite (NaNO_2_, Sigma‐Aldrich, cat. no.: 8222850100), ^1^ ^5^N‐sodium nitrite (Na^1^ ^5^NO_2_, Sigma‐Aldrich, cat. no.: 490814), sodium thiosulfate (STS, Supelco, cat. no.: 1065120250), trimethoxyvinylsilane (Sigma‐Aldrich, cat. no.: 235768), 1H,1H,2H,2H‐perfluorodecanethiol (PFDT, Sigma‐Aldrich, cat. no.: 660493), rhodamine 6G (Sigma‐Aldrich, cat. no.: R4127), 10 cSt silicon oil (Sigma‐Aldrich, cat. no.: 378321), fish gelatin from cold water fish skin (Sigma‐Aldrich, cat. no.: G7041), bovine serum albumin (BSA, Sigma‐Aldrich, cat. no.: A7906), sulfanilamide (Sigma‐Aldrich, cat. no.: S9251) and N‐(1‐naphtyl)ethylenediamine dihydrochloride (Sigma‐Aldrich, cat. no.: 222488), in vitro toxicology assay kit resazurin based (Sigma‐Aldrich, cat. no.: TOX8) were purchased from Merck KGaA (Darmstadt, Germany). Sodium ascorbate (SA, cat. no.: BD151371) was purchased from BLD Pharmatech GmbH (Reinbek, Germany).

Dulbecco's phosphate buffered saline (DPBS without calcium and magnesium, Gibco, cat. no.: 14190144), Dulbecco's Modified Eagle Medium (DMEM, Gibco, cat. no.: 41966029), 0.25% trypsin‐EDTA (Gibco, cat. no.: 25200056), fetal bovine serum (FBS, Gibco, cat. no.: A5256701), FluoroBrite DMEM (Gibco, cat. no.: A1896701), Calcein AM (Invitrogen, cat. no.: C3099), propidium iodide (PI, Invitrogen, cat. no.: J66584.AB), Hoechst 33342 trihydrochloride trihydrate (Hoechst, Invitrogen, cat. no.: H3570), formaldehyde 16% w/v (cat. no.: 28908), Goat anti‐Rabbit IgG (H+L) Secondary Antibody DyLight 488 (Invitrogen, cat. no.: 35552), and Ki‐67 polyclonal antibody (Invitrogen, cat. no.: PA5‐19462) were purchased from Thermo Fisher Scientific, Darmstadt, Germany. PhenoVue Fluor 568 ‐Phalloidin (cat. no.: CP25681) was purchased from Revvity (Hamburg, Germany). LDH‐cytotoxicity assay kit (fluorometric, cat. no.: AB197004) was purchased from Abcam (Cambridge, United Kingdom).

Ethylenediaminetetraacetic acid (EDTA, cat. no.: 20301.186), HEPES free acid (cat. no.: 0511–50G) were purchased from VWR International GmbH (Darmstadt, Germany). Silica nanoparticles AEROSIL 200 were obtained from Evonik Industries AG (Essen, Germany). Photo masks were bought from Rose Fotomasken (Bergisch Gladbach, Germany).

### 4.2 Methods

#### 4.2.1 UV/Vis Spectroscopy

Measurements were performed with a Cary 3500 Multicell spectrophotometer (Agilent Technologies, United States) using 1.4 mL quartz cuvettes with a 10 mm path length. Kinetics experiments were done with an averaging time of 0.06 s, spectral bandwidth of 2 and 1 nm data interval. For single time point experiments, the same parameters were used except the averaging time, which was 0.02 s. All spectra were recorded between 200 and 800 nm.

#### 4.2.2 NMR Spectroscopy

For ^1^H‐NMR spectra of the synthesized molecules were measured with a Bruker Ascend DRX 400 NMR spectrometer (frequency: ^1^H = 400 MHz) at room temperature. The peak shifts were declared in parts per million (ppm) and were reported relative to the solvent peak or to TMSP.

The ^15^N‐spectrum (pulse sequence zg) was recorded on a 600 MHz Bruker Avance III spectrometer equipped with a BBI room temperature probehead (Bruker BioSpin) at 26.9°C. 12288 scans with 32768 points were acquired at a spectral width of 100 ppm. Spectral frequency was set to 750 ppm. The spectrum was measured and processed using Topspin (Bruker BioSpin). An exponential window function with a line broadening factor of 20 Hz was applied to the FID after one time zero filling. Phase and baseline correction was performed manually.

#### 4.2.3 LC‐MS

LC‐MS measurements were performed on an Agilent 1260 Infinity II system consisting of a quaternary pump (GB7111B;), autosampler (G7129A, 100 µL sample loop), a temperature‐controlled column oven (G7114A) and a variable UV–vis detector (G7114 A, VWD, flowcell G7114A 018, *d* = 10 mm, *V* = 14 µL). Separation was performed on a C18 HPLC‐column (Agilent Poroshell 120 EC‐C18 4.6×100 mm, 2.7 µm) operating at 40°C. A gradient of MeCN:H2O 10:90–80:20 v/v (additive 10 mmol·l‐1 NH4HCO2) at a flow rate of 1 mL·min‑1 during 15 min was used as the eluting solvent. The flow was directed into an Agilent MSD (G6136BA, AP‐ESI ion source). The instrument was calibrated in the *m/z* range 118–2121 in the positive mode and 113–2233 in the negative using a premixed calibration solution (Agilent). The following parameters were used: Spray chamber flow: 12 L·min^−1^; drying gas temperature: 350 K, Capillary Voltage: 3000 V, Fragmentor Voltage: 100 V. Samples were prepared by mixing NEM 1 mM and SA 30 mM in a 1:1 mixture of DI water and acetonitrile and subsequently incubated for 1 h at 37°C.

#### 4.2.4 S‐Nitrosylation of Thiol‐Modified PEG

PEGSH (100 mg, 0.0400 mmol, 1.00 eq.) was dissolved in HCl 0.01 M (10 mL) in water and stirred at 0°C for 10 min. To maintain an inert atmosphere and prevent oxidative degradation, nitrogen was bubbled through the reaction mixture from this point on throughout the course of the reaction. NaNO_2_ (2.76 mg, 0.0400 mmol, 1.00 eq.) dissolved in DI water (200 µL) was added dropwise to the solution and the reaction was stirred for 30 min at 0°C. The reaction was terminated by drop wise addition of cold, degassed PBS 10x (1.1 mL). The resulting light pink solution was freeze dried to yield the desired product as a light pink solid in quantitative yield. The product was stored under vacuum protected from light at −20°C until further use. For ^1^H‐NMR analysis, PEGSNO and PEGSH were solubilized in deuterium oxide at 15 mg/mL and measured immediately. For ^15^N‐NMR, PEGSNO was solubilized also in deuterium oxide, but at 100 mg/mL and measured right away.

#### 4.2.5 Quantification of Thiols via Ellman's Assay

Thiols were quantified using Ellman's assay, which is based on the quantitative reaction of DTNB with free thiols to yield a mixed disulfide and 2‐nitro‐5‐thiobenzoic acid, a colored species whose absorbance can be measured at 412 nm. Sodium phosphate 100 mM with EDTA 1 mM pH 8 was used as the reaction buffer. For confirming the S‐nitrosylation, PEGSH was solubilized at 0.125% w/v (∼0.46 mM SH groups) in DI water and PEGSNO was solubilized at 1% w/v. For quantifying free thiols generated upon nucleophile‐triggered decomposition of PEGSNO, samples containing PEGSNO 0.25% w/v and the nucleophiles at 30 mM were incubated for 1 h at 37°C and subsequently cleaned two successive times with reaction buffer by centrifugal ultrafiltration for 50 min at 12 000 rpm at 4°C using a 3 kDa MWCO membrane (Vivaspin 500, Sartorius, Göttingen, Germany). The samples recovered from the filters were manually adjusted to the same final volume with reaction buffer. For the measurements, 1.25 mL of reaction buffer were mixed with 125 µL of the sample and 25 µL of DTNB prepared at 4 mg/mL in reaction buffer. The reaction was left to proceed for 10 min at room temperature and then the UV/Vis spectra were recorded. A calibration curve with 2‐mercaptoethanol at serial dilutions between 0.01 and 0.8 mM was used for quantification purposes (Figure S1).

#### 4.2.6 Quantification of NO‐Related Species via Griess Assay

Griess assay was used to quantify NO‐related species by measuring nitrite (NO_2_^−^) as one of the main stable breakdown products of NO in aqueous systems. The assay is based on the reaction of sulfanilamide with NO_2_^−^ in acidic conditions to yield a diazonium salt, which then reacts with NED to produce a diazo dye whose absorbance can be measured at 540 nm. Samples were prepared by mixing PEGSNO at 0.25% w/v with STS or SA at 30 mM in HEPES 25 mM with EDTA 0.1 mM pH 7.4 and incubated for 1 h at 37°C. After incubation, 100 µL of sample were combined with 1.2 mL of DI water and 50 µL of 1% w/v sulfanilamide in 5% phosphoric acid. The samples were incubated for 5 min and 50 µL of 0.1% w/v NED in DI water were added and mixed by pipetting. The UV/Vis spectra of the samples were recorded after 15 min incubation at room temperature.

#### 4.2.7 Surface Patterning of Droplet Microarrays (DMAs) on Borosilicate Glass Slides

Droplet microarrays were surface patterned on Nexterion glass slides (Schott AG, Mainz, Germany) by following a patented procedure [53]. Briefly, ozone‐activated glass slides were first spin‐coated with 500 µL of a vinyltrimethoxysilane‐functionalized silica nanoparticle solution for five consecutive times at 1000 rpm for 15 s using a SPIN150i spin coater (SPS Europe, Ingolstadt, Germany). Freshly coated slides were cured for 1 h at 150°C using a heating plate (Präzitherm PZ60, Harry Gestigheim GmbH, Düsseldorf, Germany). Next, a super hydrophobic pattern was generated by covering the slides with a 10% v/v PFDT solution in isopropanol and exposing them through a photo mask comprising a 5 × 16 array of Φ 3.2 mm circular spots to UV light for 90 s using a UVA Cube 2000 (Dr. Hönle AG, Gilching, Germany). The patterned slides were then covered with a solution of 10% v/v 2‐mercaptoethanol in ethanol/water (1:1) and irradiated again for 90 s through a quartz glass to distribute the solution evenly.

#### 4.2.8 Non‐Contact Dispensing of Hydrogel Precursors on DMA Slides

Hydrogel precursors (PEGSNO, PEGSH and PEGMAL at 6% w/v, STS and SA at 150 mM) were prepared fresh in HEPES 25 mM EDTA 0.1 mM pH 7.4 and dispensed on DMA slides using a Certus Flex (nNano AG, Gwatt, Switzerland) non‐contact liquid dispenser equipped with Φ 0.15‐ and 0.3 mm valves. The precursors were dispensed sequentially (buffer first, then SA or STS, then PEGMAL, followed by PEGSNO or PEGSH last) to a final volume of 3 µL per spot 26 different formulations in triplicates including controls (Table S3) were dispensed on each slide. After dispensing, slides were placed in a Petri dish with a humidified pad on the cap to prevent evaporation and incubated for 30 min in a CO_2_ incubator at 37°C for gelation, and then washed in warm DPBS by five consecutive immersions to remove uncrosslinked or unstable samples. Finally, the slides were submerged overnight in a rhodamine 6G solution (10 µM) at room temperature for swelling until equilibrium and imaged submerged in DPBS using a wide field fluorescence microscope (Keyenze BZ‐X800, Keyence Co. Osaka, Japan) in the TRITC channel (excitation 544 nm, emission 570 nm).

#### 4.2.9 Fluorescence Image Analysis for Gelation Window Determination and Heterogeneity Assessment

Fluorescence images of hydrogel arrays were analyzed in Fiji (ImageJ, NIH). After 8‐bit conversion and rolling‐ball background subtraction (with constant radius across the dataset), spots were segmented using a fixed threshold, followed by a morphological cleanup (Fill holes and Open). The position of the spots was related to the formulation dispensed according to the printing layout and only the formulations for which the three repetitions were stable were counted as crosslinked for the gelation window in Figure 3C. Next, Analyze Particles using size and circularity filters was applied to the images and the resulting ROIs were used to extract per‐spot mean intensity and standard deviation, specifically for three formulations: PEGSH 2% w/v + PEGMAL 2% w/v, PEGSNO 2% w/v + PEGMAL 2% w/v + STS 50 mM, and PEGSNO 2% w/v + PEGMAL 2% w/v + SA 50 mM. Two heterogeneity metrics were computed for each spot: An intra‐spot intensity coefficient of variation (CV = σ/µ) and an edge heterogeneity index, defined as the mean intensity of a Sobel‐filtered duplicate divided by the mean raw intensity within the same ROI. Spot‐level metrics were pooled across replicates and reported as mean ± SD. Differences between the PEGSH control and PEGSNO formulations were assessed on experiment‐level averages by one‐way ANOVA with Dunnett's post‐hoc test (PEGSH as reference).

#### 4.2.10 Bulk Rheology

Experiments were performed using a DHR‐30 rheometer (TA Instruments, USA) equipped with a 20 mm parallel plate geometry and a Peltier plate for temperature control. All experiments were performed with a 500 µm gap and in triplicate. For each condition, results are shown as the average of the three runs with the standard deviation as error. For the gelation kinetics experiments, PEGSNO and PEGMAL at an equimolar ratio of functional groups (8 mM or 2% w/v each, 4% w/v total) were mixed with STS or SA immediately before loading. All precursor solutions were prepared immediately before use in HEPES 25 mM EDTA 0.1 mM buffer pH 7.4 Approximately 250 µL of sample were used for each run and a few droplets of 10 cSt silicon oil were added to the borders after sample trimming to prevent evaporation during the measurements. First, an oscillatory temperature ramp (3°C/min) at 1% strain and 1 rad/s was performed between 22° and 37°C, followed by a time sweep under the same conditions at 37°C for 100 min. The apparent gelation times t_50_ and t_90_ were defined as the times required for the storage modulus G′ to reach 50% and 90% of its final plateau value, respectively, and the plateau modulus G′_ꝏ_ was taken as the mean G′ over the last 10 min of each experiment. Gelation kinetics curves were fitted with a 4‐parameter logistic function in MATLAB (R2024b, The MathWorks, Inc., Natick, MA, United States) to extract the maximum gelation rate (dG' dt^−1^).

#### 4.2.11 Characterization of the Mechanical Properties of Swollen Hydrogels

7 uL of hydrogel precursor solution were pipetted into 6‐well microtiter plates, incubated for 30 min at 37°C and subsequently washed three times with warm DPBS. The crosslinked samples were left to swell in DPBS overnight and measured submerged in fresh DPBS at room temperature. Measurements were performed with a Pavone nanoindenter (Optics11 Life, Amsterdam, Netherlands) using a probe with cantilever stiffness 0.42 N m^−1^ and a spherical tip with radius 27.5 µm. For evaluating the compressive stiffness, that is, Young's modulus, the peak‐load poking mode with a maximum load of 0.1 µN and piezo speed of 10 µm s^−1^ was used.

The mechanical properties were obtained from the measurements using the Proba software (Optics11 Life, Amsterdam, Netherlands). In particular, the Hertz model with Pmax set at 80% was used to extract the Young's modulus, limiting the fitting of the data to a maximum indentation depth corresponding to 16% of the tip radius. Measurements where the R^2^ of the fit fell below 0.95 or where the measurement started in contact were discarded for analysis.

#### 4.2.12 Raman Spectroscopy Measurements

Raman spectra were recorded on a Bruker Senterra 2 spectrometer (Bruker Optics, Ettlingen, Germany) with 785 nm excitation laser operated at 50 mW power. For sample observation, focusing of the laser and back collimation, an Olympus MPLAN 20x NA 0.4 objective was used. Spectra were recorded with a spectral resolution of 4 cm^−^^1^, integration time was 40 s with eight coadditions (8*5 s) per spectrum, and bandwidth was 50–3640 cm^−^^1^.

#### 4.2.13 Synthesis of Maleimide‐Modified Cold Water Fish Skin Gelatin (GelfMAL)

GelfMAL was synthesized based on a previously reported protocol [54]. Briefly, 1.5 g of fish gelatin (0.236 mmol lysines, 1 eq.) was solubilized in a mixture of 30 mL of DI water and 25 mL of DMSO under constant magnetic stirring at 40°C. Next, 0.627 g N[(3‐maleimidopropionyl)oxy]succinimide (2.36 mmol, 10.0 eq.) were solubilized in 5 mL DMSO and added drop wise to the solubilized gelatin. The mixture was then left to react under constant magnetic stirring at 40°C for 24 h. The resulting solution was diluted with the same volume of DI water and dialyzed (12–14 kDa MWCO) against DI water for 4 d with three water changes per day. The dialyzed product was then freeze dried and stored in sealed containers under vacuum at −20°C until further use. The degree of functionalization of GelfMAL was estimated via ^1^H‐NMR spectroscopy. Pristine fish gelatin and GelfMAL were solubilized at 15 mg/mL in deuterium oxide and TMSP 0.2% w/v was added as an internal standard for quantifying the extent of the modification.

#### 4.2.14 Cell Culture

NIH/3T3 mouse embryonic fibroblasts and A549 human alveolar adenocarcinoma epithelial cells were maintained in complete growth medium consisting of DMEM supplemented with 10% FBS at 37°C in a CO_2_ incubator. Cells were passaged at ∼80% confluency using 0.25% trypsin‐EDTA and used between passages 20–29. Cytotoxicity of the nucleophiles towards both cell lines was evaluated with a resazurin‐based metabolic viability assay (TOX8) and an LDH‐release membrane integrity assay. Cells were harvested and seeded in 96‐well microplates at 15,000 cells/cm^2^ and allowed to attach overnight in complete growth medium (37 °C, 5% CO_2_). The next day, the medium was replaced with nucleophile solutions prepared in serum‐free DMEM containing EDTA 0.1 mM (added to suppress side reactions) over concentrations ranging between 0.938 and 100 mM, and cells were exposed for 30 or 120 min. The solutions were then removed, replaced with complete growth medium and returned to incubation. After 24 h, LDH release was quantified in the culture supernatant and metabolic activity in the remaining adherent cells, each according to the respective manufacturer’s protocol. Untreated cells provided the viability reference, wells lysed with the kit’s lysis buffer the maximum LDH release control, and medium‐only wells the background. Both assays were measured fluorometrically with a CLARIOstar Plus microplate reader (BMG LABTECH, Ortenberg, Germany) considering the manufacturer’s instructions for the excitation and emission wavelengths of each assay.

#### 4.2.15 Cell Encapsulation in PEGSNO/GelfMAL Hydrogels

Hydrogel precursor solutions were prepared in a buffer containing NaCl 150 mM, HEPES 25 mM, EDTA 0.1 mM pH 7.4. Three formulations were tested: PEGSNO 2% w/v + GelfMAL 4.7% w/v + STS 50 mM, PEGSNO 3% w/v + GelfMAL 7% w/v + STS 30 mM, and PEGSNO 3% w/v + GelfMAL 7% w/v + STS 50 mM. Immediately after mixing the components, trypsinized cells were resuspended in the hydrogel precursor solution at 5 × 10^5^ cells mL^−1^ and 7 µL droplets were pipetted into four well multidishes. The cell‐embedded hydrogels were incubated inverted for 30 min in a CO_2_ incubator at 37°C and subsequently washed thrice with warm DPBS. The constructs were then maintained in complete growth medium for up to 72 h without any medium exchange before viability or immunostaining analysis.

#### 4.2.16 Live/Dead Staining and Image Analysis

After one or three days of incubation, medium was removed from the wells and replaced with a staining solution consisting of Calcein‐AM (1:2000, 0.5 µM), PI (1:500, 3 µM), and Hoechst (1:2000) in FluoroBrite DMEM. Samples were incubated for 30 min in a CO_2_ incubator at 37°C and imaged with a Keyence BZ‐X800 fluorescence microscope at 10× magnification using the sectioning mode. ∼100 µm z‐stacks with a 10 µm pitch were acquired for each hydrogel, and at least three hydrogels were imaged per formulation, per timepoint. Maximum‐intensity projection images for each channel (blue for Hoechst, red for PI and green for Calcein AM) were analyzed with a custom‐made pipeline in CellProfiler (version 4.2.8) [55]. Viability was reported as the percentage of Calcein AM‐positive cells over total cells (Calcein AM‐positive; PI‐positive, and double positive). Only Hoechst‐positive cells were considered for analysis.

#### 4.2.17 Immunostaining

At day 3, hydrogels were rinsed with DPBS, fixed in 4% w/v formaldehyde in DPBS for 30 min at room temperature, washed thrice with DPBS for 5 min under mild agitation and stored in fresh DPBS at 4°C until staining. Samples were permeabilized with 0.3% v/v Triton X‐100 in DPBS for 30 min at room temperature, washed thrice with DPBS for 5 min under mild agitation and blocked with a solution of 5% w/v BSA (blocking buffer) for 1 h at room temperature. Next, samples were incubated overnight at 4°C with rabbit anti‐Ki‐67 primary antibody in blocking buffer and then with goat anti‐rabbit Alexa Fluor 488 (1:500, 2 µg/mL) and PhenoVue 568 phalloidin (1:400, 165 nM) for 2 h at room temperature under constant mild agitation and protected from light. Nuclei were stained with DAPI in blocking buffer for 15 min at room temperature and the samples were washed twice with DPBS for 5 min before imaging. ∼100 um z‐stacks with a 1 µm pitch were acquired with a Leica Thunder fluorescence microscope (Leica Microsystems GmbH, Wetzlar, Germany) using either 20x or 40x objectives.

#### 4.2.18 Statistical Analysis

All quantitative data are presented as mean ± standard deviation, with the number of independent replicates (n) specified in the corresponding figure captions. Statistical comparisons were performed in MATLAB (R2024b, The MathWorks, Inc., Natick, MA, United States). Differences between two groups were assessed using two‐sample Student's t‐tests. Comparisons across more than two groups were performed using one‐way analysis of variance (ANOVA) followed by Tukey's honestly significant difference (HSD) or Tukey‐Kramer post‐hoc tests for pairwise comparisons. For experiments involving two factors (e.g., nucleophile identity and concentration) two‐way ANOVA was used. Differences were considered statistically significant at *p* < 0.05.

#### 4.2.19 Use of large language models (LLMs)

Claude (Anthropic, model versions Sonnet 4.6 and Opus 4.7) were used for proofreading the manuscript text. The LLM was not used for data analysis, figure generation, or formulation of scientific conclusions. All AI‐assisted output was reviewed, edited, and verified by the authors.

## Conflicts of Interest

The authors declare no conflicts of interest.

## Supporting information




**Supporting File 1**: adma74627‐sup‐0001‐SuppMat.docx.

**Supporting File 2**: adma74627‐sup‐0002‐MovieS1.mp4.

**Supporting File 3**: adma74627‐sup‐0003‐MovieS2.mp4.

**Supporting File 4**: adma74627‐sup‐0004‐MovieS3.mp4.

## Data Availability

The data that support the findings of this study are available from the corresponding author upon reasonable request.
