# Supplementary material for: S‐Nitrosothiols as Thiol‐Protecting Groups for Controlled Thiol‐Maleimide Crosslinking of Homogeneous Soft Hydrogels
Source: Adv Mater. 2026 Aug 17;38(57):e74627. doi: 10.1002/adma.74627 (PMC13660815; doi:10.1002/adma.74627)
Supplement: Supplementary file 1 [file ADMA-38-e74627-s001.docx]

**Supplementary information file**

**S-Nitrosothiols as Thiol‑Protecting Groups for Controlled Thiol‑Maleimide Crosslinking of Homogeneous Soft Hydrogels**

Julian A. Serna^1^, Michelle J. Iwohn^1^, Maximilian Seifermann^1^, Stefan Heissler^2^, Maike Schliephake^1^, Franziska Dopp^2^, Claudia Muhle-Goll^2^, Pavel A. Levkin*^1,3^

^1^ Institute of Biological and Chemical Systems-Functional Molecular Systems (IBCS-FMS), Karlsruhe Institute of Technology (KIT), Kaiserstraße 12, 76131 Karlsruhe, Germany

^2^ Institute of Functional Interfaces (IFG), Karlsruhe Institute of Technology (KIT), Kaiserstraße 12, 76131 Karlsruhe, Germany

^3^ Institute for Biological Interfaces 4 (IBG-4), Karlsruhe Institute of Technology (KIT), Kaiserstraße 12, 76131 Karlsruhe, Germany

^4^ Institute of Organic Chemistry (IOC), Karlsruhe Institute of Technology (KIT), Kaiserstraße 12, 76131 Karlsruhe, Germany

Corresponding author: [levkin@kit.edu](mailto:levkin@kit.edu)

**Figure S1.** Calibration curve of Ellman’s assay with varying concentrations of 2‑mercaptoethanol (0.01 – 0.8 mM). Each data point corresponds to the mean of the absorbance at 412 nm with the standard deviation as error.

**Table S1.** Quantification of the degree of S‑nitrosylation with Ellman’s assay. The calibration curve was done with 2‑mercaptoethanol (**Figure S1**) and the unmodified thiol-PEG (PEGSH) was used as a positive control for thiol quantification.

| **Sample** | **Concentration (% w/v)** | **Theoretical thiol concentration before modification (mM)** | **Absorbance at 412 nm (n=3) (mean +/- std)** | **Estimated thiol concentration (mM)** | **Degree of modification (%)** |
| --- | --- | --- | --- | --- | --- |
| PEGSH 4arm 10 kDa | 0.125 | 0.4750 | 0.5998 +/- 0.0004 | 0.4608 | - |
| PEGSNO 4arm 10 kDa | 1 | 3.8000 | 0.0143 +/- 0.1115x10^3^ | < 0.0125 | > 99% |

**Figure S2.** (A) Decay kinetics at 37 °C of the 545 nm peak of PEGSNO by the addition of sodium ascorbate (SA) and (B) its fit to a first-order model.

**Table S2.** Parameters of the pseudo-first order fit of the decomposition of PEGSNO with SA (**Figure S2B**).

| **PEGSNO 1 mM RSNO + SA 30 mM** | | | |
| --- | --- | --- | --- |
|  | ***k*** | **t/2 (s)** | **R^2^** |
| Run 1 | 4.72x10^-4^ | 1.47x10^3^ | 0.9968 |
| Run 2 | 4.09x10^-4^ | 1.70x10^3^ | 0.9986 |
| Run 3 | 5.23x10^-4^ | 1.32x10^3^ | 0.9938 |
| Mean | 4.6817x10^-4^ | 1.4957x10^3^ |  |
| SD | 5.7250x10^-5^ | 1.8672x10^2^ |  |

**Figure S3.** (A) UV/Vis kinetics at 37 °C of the 545 nm peak of PEGSNO by the addition of sodium thiosulfate (STS) and (B) its fit to a first-order model.

**Figure S4.** Quantification of regenerated thiols via Ellman’s assay. Absorbance at 412 nm was measured following a 1 h incubation (1 mM RSNO/SH, STS/SA 30 mM) and subsequent centrifugal ultrafiltration (MWCO 3 kDa). PEGSH was used as a positive control for total thiol recovery, confirming minimal oxidation or loss during purification, and PEGSNO alone as a negative control for spontaneous decomposition. Experimental groups represent PEGSNO treated with nucleophiles (STS or SA). Horizontal reference lines indicate background interference from nucleophile solutions (lacking PEGSNO) subjected to identical incubation and purification steps: red dashed line (SA control) and red dotted line (STS control). Data represent means and error bars correspond to standard deviation (n=3).

**Figure S5.** UV/Vis kinetics of SA 30 mM alone at 37 °C.

**Figure S6.** ^1^H-NMR spectra confirmed consumption of N-ethylmaleimide (NEM) in the presence of sodium ascorbate (SA). Top: ¹H-NMR spectrum of NEM in D₂O, showing the characteristic maleimide vinylic signal at ~6.9 ppm (green), the N‑CH₂ signal at ~3.5 ppm (pink), and the terminal CH₃ signal at ~1.1 ppm (yellow). Bottom: ¹H-NMR spectrum of a NEM + SA mixture (1:3 molar ratio) after 1 h of incubation at 37 °C in D₂O. The maleimide vinylic signal at ~6.9 ppm was absent, while the aliphatic NEM signals are retained in shifted positions, consistent with addition across the maleimide double carbon bond.

**Figure S7**. LC‑MS analysis of an N-ethylmaleimide (NEM) and sodium ascorbate (SA) mixture confirms formation of an ascorbate‑maleimide adduct. UV chromatogram (λ = 254 nm) of a 1 mM NEM / 30 mM SA solution after 1 h of incubation at 37 °C, showing a single dominant peak at a retention time of 1.21 min (left). Inset: ESI mass spectrum acquired at the apex of the chromatographic peak, with the principal ion at m/z 302.050 corresponding to the ascorbate‑maleimide Michael adduct, accompanied by its sodium ([M+Na]⁺, 324.050) and likely dimer-related ([2M+H]⁺, 621.900) species.

**Figure S8.** ¹H-NMR spectra confirmed consumption of N-ethylmaleimide (NEM) in the presence of sodium thiosulfate (STS). Top: ¹H-NMR spectrum of NEM in D₂O, showing the characteristic maleimide vinylic signal at ~6.9 ppm (green), the N‑CH₂ signal at ~3.5 ppm (pink), and the terminal CH₃ signal at ~1.2 ppm (yellow). Bottom: ¹H-NMR spectrum of a NEM + STS mixture (1:3 molar ratio) after 1 h of incubation at 37 °C in D₂O. The maleimide vinylic signal at ~6.9 ppm was absent, while the aliphatic NEM signals are retained in shifted and broadened positions, consistent with addition of thiosulfate across the maleimide double carbon bond to form the corresponding NEM‑S‑thiosulfate (Bunte salt) adduct.

**Figure S9.** (A) UV/Vis kinetics of the interaction between STS (30 mM) and NEM (1 mM) in DPBS, HEPES 25 mM, sodium phosphate buffer 25 mM, and sodium phosphate buffer 100 mM (from left to right). (B) UV/Vis kinetics of the same system after the addition of 2‑mercaptoethanol (ME) equimolar to NEM.

**Table S3**. Formulations dispensed on the DMA to screen for the formation of stable hydrogels at different polymer concentrations, nucleophile type (SA or STS) and nucleophile concentration.

| **Formulation ID** | **PEGSNO (% w/v)** | **PEGMAL (% w/v)** | **PEGSH (% w/v)** | **STS/SA (mM)** |
| --- | --- | --- | --- | --- |
| 1 | 1 | 1 | 0 | 5 |
| 2 | 1 | 1 | 0 | 15 |
| 3 | 1 | 1 | 0 | 30 |
| 4 | 1 | 1 | 0 | 50 |
| 5 | 1.25 | 1.25 | 0 | 5 |
| 6 | 1.25 | 1.25 | 0 | 15 |
| 7 | 1.25 | 1.25 | 0 | 30 |
| 8 | 1.25 | 1.25 | 0 | 50 |
| 9 | 1.5 | 1.5 | 0 | 5 |
| 10 | 1.5 | 1.5 | 0 | 15 |
| 11 | 1.5 | 1.5 | 0 | 30 |
| 12 | 1.5 | 1.5 | 0 | 50 |
| 13 | 1.75 | 1.75 | 0 | 5 |
| 14 | 1.75 | 1.75 | 0 | 15 |
| 15 | 1.75 | 1.75 | 0 | 30 |
| 16 | 1.75 | 1.75 | 0 | 50 |
| 17 | 2 | 2 | 0 | 5 |
| 18 | 2 | 2 | 0 | 15 |
| 19 | 2 | 2 | 0 | 30 |
| 20 | 2 | 2 | 0 | 50 |
| 21 | 2 | 0 | 0 | 50 |
| 22 | 0 | 2 | 0 | 50 |
| 23 | 0 | 1 | 1 | 0 |
| 24 | 0 | 1.25 | 1.25 | 0 |
| 25 | 0 | 1.5 | 1.5 | 0 |
| 26 | 0 | 2 | 2 | 0 |

**Table S4**. Kinetic parameters extracted from four-parameter logistic fits of rheological gelation curves for PEGSNO/PEGMAL hydrogels (2% w/v each) crosslinked with STS or SA at 30 or 50 mM. G'ₘₐₓ: storage modulus at the plateau of the fitted curve. t₁/₂: time to reach half-maximal G'. K: characteristic timescale of the logistic fit. dG' dt^-1^ at t₁/₂: maximum rate of stiffening, evaluated at the inflection point. R²: goodness-of-fit of the logistic model. Values are mean ± standard deviation across 3 independent replicates.

|  | **PEGSNO 2% w/v + PEGMAL 2% w/v** | | | |
| --- | --- | --- | --- | --- |
|  | **STS** | | **SA** | |
|  | **30 mM** | **50 mM** | **30 mM** | **50 mM** |
| G’_max_ (Pa) | 416 ± 77 | 520 ± 43 | 231 ± 20 | 310 ± 43 |
| t_1/2_ (min) | 46.0 ± 3.5 | 27.3 ± 0.9 | 99.7 ± 2.2 | 57.2 ± 1.6 |
| K (s) | 310 ± 16 | 192 ± 6.9 | 1081 ± 19 | 754 ± 14 |
| dG’ dt^-1^ at t_1/2_ (Pa/s) | 0.34 ± 0.06 | 0.68 ±0.05 | 0.053 ± 0.006 | 0.10 ± 0.02 |
| R^2^ | > 0.99 | > 0.99 | > 0.99 | > 0.99 |

**Figure S10.** Individual rheological and axial-force traces for the PEG-based hydrogel formulations. Storage modulus G’ and loss modulus G’’ and the corresponding axial force were recorded during oscillatory time sweeps at 37 °C under a fixed 500 µm gap. Each line represents one independent replicate. Circles in the axial-force plots indicate the force measured at the time of a local maximum in G’. Negative axial-force values indicate tensile force consistent with contraction against the fixed-gap geometry. A reproducible post-maximum decline in G’ was most pronounced for STS 50 mM and was smaller for STS 30 mM, whereas the SA 50 mM did not reach a clear terminal maximum within the measurement window.

**Figure S11.** Raman spectroscopic evidence of maleimide consumption and STS involvement during gelation of the PEG-based system (PEGMAL 4% w/v, PEGSNO 4% w/v and STS 50 mM). Raman spectra (785 nm excitation, confocal), normalized to the PEG backbone band at 848 cm⁻¹ (C–O/C–C). Bottom: full-range spectra (100–3600 cm⁻¹); the two dashed boxes mark the regions expanded above. Top left (400–1050 cm⁻¹), thiosulfate and PEG-backbone region: dashed guides indicate the thiosulfate S–S stretch (447 cm⁻¹) and symmetric SO₃ stretch (997 cm⁻¹) of STS, and the PEG backbone normalization band (848 cm⁻¹). Top right (1350–2000 cm⁻¹), maleimide region: dashed guides indicate the maleimide C=C stretch (1588 cm⁻¹) and C=O (imide carbonyl) stretch (1773 cm⁻¹). Traces, top to bottom: STS 50 mM; PEGMAL 4% w/v; the PEGMAL 4% w/v + PEGSNO 4% w/v + STS 50 mM gel at T = 0, 15, and 30 min after mixing and incubating at 37 °C; and PEGSH 4% w/v (maleimide-free thiol reference, control). The progressive attenuation of the maleimide C=C (1588 cm⁻¹) and C=O (1773 cm⁻¹) bands from PEGMAL through the T = 0 → 15 → 30 min series toward the PEGSH floor indicates consumption of maleimide groups by thiol–maleimide Michael-type addition and formation of thiosuccinimide (thioether) linkages; the thiosulfate bands (447, 997 cm⁻¹) confirm STS presence in the reacting mixture. Spectra are offset vertically for clarity.

**Figure S12**. ¹H-NMR characterization of maleimide-modified cold water fish skin gelatin (GelfMAL). Top: ¹H-NMR spectra of unmodified gelatin (Gelf) and GelfMAL (1:10 modification ratio) in D₂O, with TMSP as internal reference (set to 9.00 protons). Bottom: zoomed-in view of the 6.4–8.0 ppm region, highlighting the maleimide vinylic signal at ~6.9 ppm (green), which is present in GelfMAL and absent in unmodified Gelf. Integration of this signal relative to the TMSP reference (0.44) yields a degree of modification of 0.170 mmol maleimide per gram of gelatin.

**Figure S13**. Gelation rate and plateau modulus of the cell-encapsulation formulations. (A) Gelation rate (dG′ dt^-1^) of PEGSNO_2_STS_50_, PEGSNO_3_STS_30_, and PEGSNO_3_STS_50_, obtained from the time derivative of a logistic fit to the oscillatory rheology gelation kinetics (Figure 5D, main manuscript). The peak height and position reflect the maximum crosslinking rate and its timing: PEGSNO3STS50 gels fastest, PEGSNO3STS30 is intermediate, and PEGSNO2STS50 shows the lowest, latest rate peak. (B) Storage modulus at the plateau (G′_max_) of the same formulations, determined directly from the gelation kinetics experiments, showing that G′_max_ increases with PEGSNO content and STS concentration. Data in (B) are shown as mean ± SD, n = 3 (* p < 0.05, *** p < 0.001, one-way ANOVA with Tukey-Kramer post-hoc tests).

**Figure S14**. Metabolic activity of A549 human lung carcinoma epithelial cells in monolayer measured by a resazurin reduction assay after 30 and 120 min of exposure to sodium thiosulfate (STS) (left panel) across concentrations spanning 0.9-100 mM. LDH release from A549 cells under the same exposure conditions, expressed as membrane damage relative to the lysis-positive control. Metabolic activity remained near control levels up to 50 mM STS, and membrane damage stayed low across the full range, confirming that the nucleophile itself is not a source of acute cytotoxicity in this cell line. Data are shown as mean ± SD, n = 4.

**Figure S15**. Representative live/dead fluorescence images of A549 human alveolar adenocarcinoma epithelial cells embedded in GelfMAL-PEGSNO-STS hydrogels. Cells were stained with calcein-AM (green, live cells), propidium iodide (red, dead cells) and Hoechst (blue, nuclei) at day 1 and day 3 post-encapsulation. Cells double stained with calcein AM and propidium iodide were counted as dead. Images for PEGSNO_2_STS_50_ were taken close to the hydrogel-media interface, since few-to-none cells were dispersed in 3D due to settling during gelation of the hydrogels. Conversely, images for the other two formulations were taken from the center of the hydrogel, which evidenced spatial distribution of the cells due to faster gelation kinetics compared to PEGSNO_2_STS_50_.

**Movie S1**. Z-stack of a ~100 µm section with a 10 µm pitch close to the hydrogel-medium interface of a representative sample of A549 cells embedded in the PEGSNO_2_STS_50_ formulation after 1 day in culture. Cells were mostly located at the boundaries of the gel due to settling before network percolation during the gelation incubation.

**Movie S2**. Z-stack of a ~100 µm section with a 10 µm pitch in the inside (approximately 100 µm from the bottom of the plate) of a representative sample of A549 cells embedded in the PEGSNO_3_STS_30_ formulation after 1 day in culture. Cells were distributed in 3D throughout the geometry of the hydrogels, indicating network percolation before cell sedimentation during the incubation period.

**Movie S3**. Z-stack of a ~100 µm section with a 10 µm pitch close to the hydrogel-medium interface of a representative sample of A549 cells embedded in the PEGSNO_3_STS_50_ formulation after 1 day in culture. Cells were distributed in 3D throughout the geometry of the hydrogels, indicating network percolation before cell sedimentation during the incubation period.
